# Decalcified and Decellularized Bone Conduits for Peripheral Nerve Regeneration: A Comparative In Vivo Study Using Rat Femur and Chicken Ulna Conduits

**DOI:** 10.1002/brb3.71546

**Published:** 2026-09-04

**Authors:** Zahra Sepehri, Ali Moradi, Nasser Mahdavi‐Shahri, Morteza Behnam Rassouli

**Affiliations:** ^1^ Department of Biology, Faculty of Science Ferdowsi University of Mashhad Mashhad Iran; ^2^ The Clinical Research Development Unit, Ghaem Hospital Mashhad University of Medical Sciences Mashhad Iran; ^3^ Orthopedic Research Center Mashhad University of Medical Sciences Mashhad Iran

**Keywords:** bone‐derived nerve guidance conduit, decellularization, demineralization, nerve guidance conduit, peripheral nerve regeneration

## Abstract

**Purpose:**

Peripheral nerve injuries (PNIs) pose a major clinical challenge, especially when nerve gaps exceed 5 mm and spontaneous regeneration is inadequate. This study aimed to evaluate the regenerative potential of novel bone‐derived nerve guidance conduits (NGCs) fabricated from rat femoral diaphysis and chicken ulna as alternatives to conventional autologous and allogeneic grafts.

**Methods:**

Demineralized and decellularized bone scaffolds were characterized for collagen preservation, luminal geometry, and mechanical and electrical properties. In vivo, the conduits were implanted to bridge 10 mm sciatic nerve gaps in rats. Functional recovery was assessed over 16 weeks using sciatic functional index (SFI), hot‐plate latency, and gastrocnemius muscle mass ratio. Histological analysis and RT‐qPCR profiling of ECM‐related genes (COL1A1, FN1, LAMB2, and Tenascin‐C) were performed to evaluate tissue integration and molecular responses.

**Findings:**

Chicken‐derived conduits showed superior tensile strength and elasticity, while both scaffold types supported hydration‐dependent ionic conductivity. In vivo results demonstrated progressive conduit resorption and nerve tissue replacement. Treated groups showed significant motor and sensory recovery compared to axotomy controls. Histology revealed early axonal infiltration, neovascularization, and remyelination. Gene expression analysis indicated scaffold‐specific temporal regulation aligned with regenerative phases.

**Conclusion:**

Bone‐derived NGCs from rat femur and chicken ulna effectively supported structural, electrophysiological, and functional nerve regeneration. These findings highlight their promise as accessible, biocompatible alternatives to traditional grafts and provide a foundation for future translational research in peripheral nerve repair.

## Graphical abstract

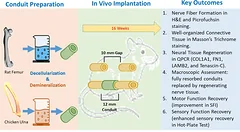

## 1 Introduction

Peripheral nerve injuries (PNIs), resulting from axonal transection or crush trauma, lead to significant motor and sensory deficits and present a major clinical challenge (Lopes et al. 2022). While peripheral nerves possess a greater intrinsic capacity for regeneration than the central nervous system, gaps exceeding 5 mm typically fail to achieve functional reinnervation without surgical intervention (Jiang et al. 2010; Singh et al. 2022). Autologous nerve grafting is considered the gold standard due to its excellent biocompatibility and minimal immunogenicity. However, its application is limited by donor‐site morbidity, restricted availability, and the requirement for a second surgical procedure (Gao et al. 2015; Mackinnon 1989; Moore et al. 2011; Dodla and Bellamkonda 2006). Allografts circumvent the need for donor harvesting but necessitate immunosuppression, thereby increasing the risk of infection and systemic complications (Anderson et al. 2008).

These limitations have spurred the development of tissue‐engineered nerve guidance conduits (NGCs) as innovative scaffolds for peripheral nerve repair (Sarker et al. 2018). NGCs, fabricated from synthetic polymers, natural proteins, or composites, aim to mimic key extracellular matrix (ECM) features, such as aligned collagen fibrils and micro‐porosity, to promote directional axonal growth (Shi et al. 2023; Yun et al. 2024; Zhang et al. 2022). An ideal NGC must offer mechanical strength suitable for suturing, exhibit biocompatibility, facilitate neurotrophic factor delivery, and promote neovascularization to sustain regenerating tissue (Yan et al. 2022; Carvalho et al. 2021). Additionally, combining NGCs with electrical stimulation has demonstrated potential in enhancing axonal regeneration and improving functional outcomes (ElAbd et al. 2022; Zarrintaj et al. 2020).

Building on these advances, we propose a novel approach utilizing demineralized, decellularized bone conduits derived from rat femoral diaphysis and chicken ulna. These naturally porous scaffolds possess inherent architecture and mechanical durability, making them well‐suited for nerve guidance. This study evaluates their in vivo performance using a 10‐mm sciatic nerve gap model in rats.

## 2 Materials and Methods

### 2.1 Animals

Eighty adult male Wistar rats (200–250 g) were housed under standard laboratory conditions (12 h light/dark cycle, 22 ± 1°C, 55 ± 5% humidity), with ad libitum access to water and chow. All procedures adhered to the ethical guidelines of Ferdowsi University of Mashhad and were approved under protocol number IR.UM.REC.1403.056.

### 2.2 Groups

Rats were randomly assigned to five experimental groups (*n* = 16 per group):

- Sham: Control group undergoing no surgical intervention.
- Axotomy (AX): Sciatic nerve transection creating a 10 mm defect without repair.
- Nerve allograft (NA): Repair with a decellularized 10 mm rat nerve segment.
- Rat bone allograft (BA): Repair using a 12 mm decalcified, decellularized rat femur diaphysis.
- Chicken bone xenograft (BX): Repair using a 12 mm decalcified, decellularized chicken ulna.

### 2.3 Preparation and Demineralization

Bones (rat femur diaphysis and chicken ulna) were initially fixed in 10% formalin for 48 h. Subsequently, they underwent bleaching using 5% sodium hypochlorite (NaOCl) followed by 5% hydrogen peroxide (H_2_O_2_) for 7 h each. Demineralization was performed using a solution of ethanol–nitric acid–formalin (ratio 20:1:2 v/v ethanol:nitric acid:formalin) until the samples became pliable. Finally, the samples were neutralized in 5% sodium sulfate for 24 h (Abedin et al. 2018; Hatta et al. 2014).

### 2.4 Decellularization

The bone conduits and rat sciatic nerve segments (20 mm lengths) were decellularized. This process involved incubating the channels and nerves sequentially in deionized water (7 h), a 1% Triton X‐100 solution (12 h), and a 4% sodium deoxycholate solution (24 h). This entire cycle was repeated twice (Lupon et al. 2025; Syazwani et al. 2015). Following decellularization, the samples were washed thoroughly and then stored in phosphate‐buffered saline (PBS) containing penicillin/gentamicin (pH 7.2) at 4°C.

### 2.5 Histology and Elemental Analysis

Decellularization efficacy was assessed using hematoxylin–eosin and DAPI staining. Demineralization was evaluated by Alizarin Red staining and energy‐dispersive X‐ray spectroscopy (EDX). Following lyophilization, sections were gold‐coated and imaged by scanning electron microscopy (SEM) to examine microarchitecture and measure inner/outer diameters and wall thickness. (Gong et al. 2022; Jiang et al. 2013).

### 2.6 Biocompatibility Evaluation of Bone Conduits (MTT Assay)

Tube‐like decellularized bone conduits (length: 5 mm) were washed with PBS containing 1% penicillin/streptomycin and then sterilized using plasma. Subsequently, the lumen of each conduit was pre‐wetted with 40 µL of working medium (DMEM‐HG, 10% FBS, and 1% Penicillin/Streptomycin). A cell suspension (2 × 10^5^ rat mesenchymal stem cells/mL) of 20 µL was then injected into each conduit lumen (*n* = 5). Following injection, the constructs were incubated for eight hours, with 2 h of incubation on each quarter side. Three identical bone conduits were prepared as non‐seeded blanks. Finally, the seeded conduits were transferred into wells of a non‐treated 48‐well plate containing 200 µL of working medium per well and incubated for an additional day.

The following day, the medium was removed, and the constructs were gently washed with pre‐warmed PBS. Subsequently, 1 mL of MTT solution (5 mg MTT powder dissolved in 1 mL PBS) was added to each well and incubated for 3 h. After removing the MTT solution, 200 µL of DMSO was added to dissolve the formazan crystals. The optical density was measured at 570 nm using a microplate reader (SpectraMax ABS Plus, Molecular Devices). Control measurements were performed using acellular bone conduits as blanks, and all experiments were conducted in triplicate (Mosmann 1983; van Tonder et al. 2015).

### 2.7 Testing

Healthy nerve and demineralized–decellularized bone conduits (30 mm total length, 20 mm gauge length; *n* = 5) were subjected to uniaxial tension at 0.1 mm/s using an STM20 (SANTAM). Young's modulus was derived from the slope of the linear region of the stress–strain curves (Borschel et al. 2003).

### 2.8 Electrical Conductivity

Dry and PBS‐hydrated conduits were measured with a two‐point probe (Seild GmbH) under varying voltages. Resistance was calculated via Ohm's law (*R* = *V*/*I*) and conductivity (*σ*) using a cylindrical model (*σ* = *L*/(*R*·*A*)) (Gong et al. 2022).

### 2.9 Surgical Procedure and Grafting

**FIGURE 1:**
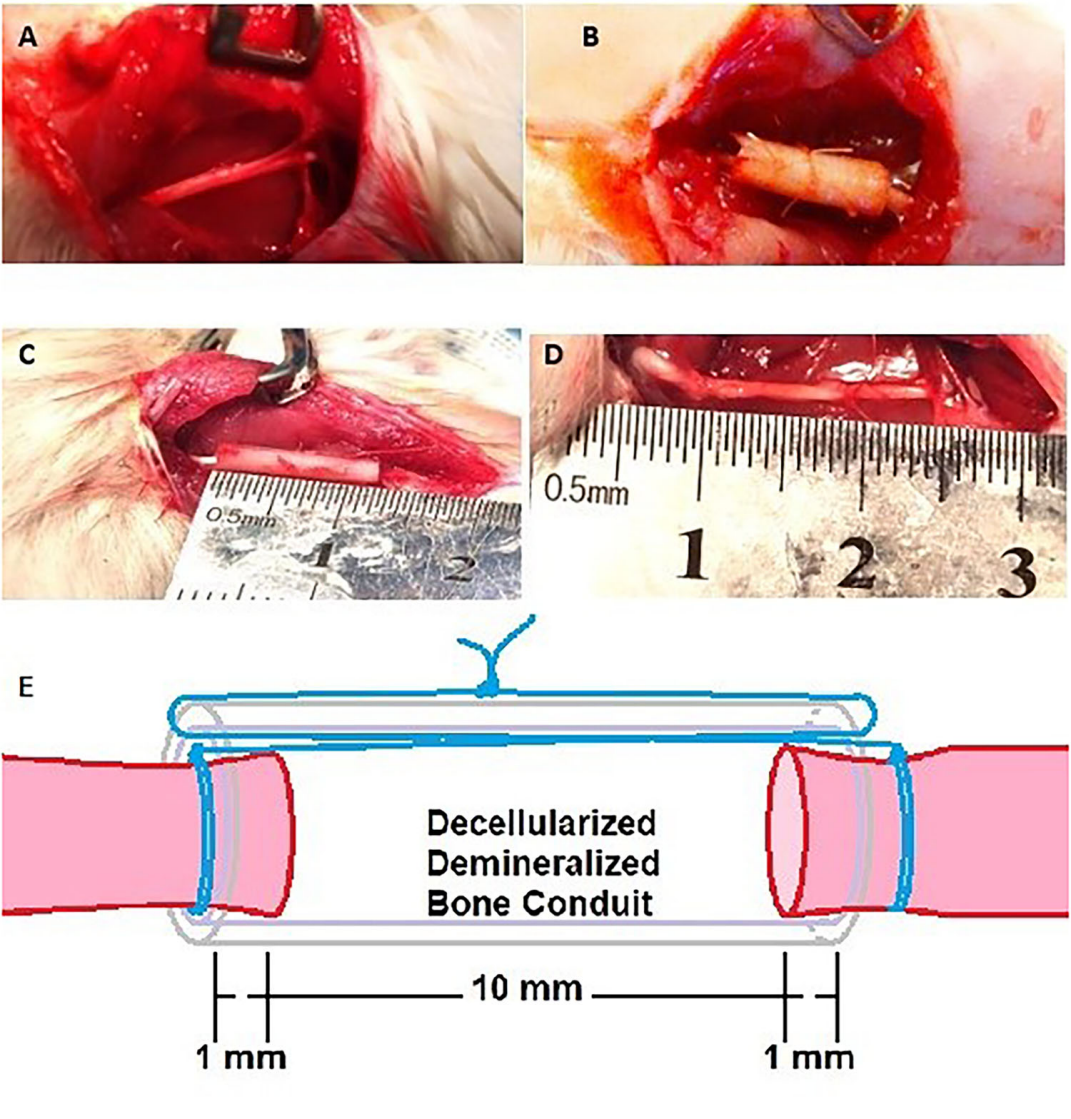
(A) Native sciatic nerve, (B) Chicken bone conduit xenograft, (C) Rat bone conduit allograft, (D) Decellularized nerve allograft, and (E) The schematic view of fixing the proximal and distal ends of nerve void inside a demineralized decellularized bone conduit.

Under intraperitoneal ketamine (50 mg/kg) and xylazine (20 mg/kg), the right sciatic nerve was exposed, which was measured as ≈1.5–2 mm in diameter. A 10 mm segment of the sciatic nerve was excised. Conduits or allografts (12 mm) were sutured in place by inserting 1 mm of each nerve stump into the conduit lumen (diameter: ≈4.5 and ≈3 mm in rat and chicken, respectively) and securing with 8‐0 nylon, leaving a 10 mm gap for regeneration (Figure 1). The site was disinfected with 70% ethanol and treated with 0.5% picric acid to prevent autotomy (Rahimi‐Movaghar et al. 1970).

### 2.10 Functional and Histological Evaluations

- **Walking Track & SFI**: Functional ambulation was assessed using the walking track test and the sciatic functional index (SFI), calculated based on footprint parameters (Bain et al. 1989).
- **Hot‐plate test**: Thermal latency was measured by placing animals on a hot plate maintained at 55 ± 1°C, with a cutoff latency of 12 s to prevent tissue damage (Hargreaves et al. 1988).
- **Muscle mass ratio**: At week 16 post‐operation, the gastrocnemius muscle mass of the operated limb was weighed and compared to the contralateral control limb to determine the muscle mass ratio (Summa Di et al. 2011).
- **Histology**: Tissues were stained using Hematoxylin & Eosin (H&E) for general structure, Masson's Trichrome for collagen, Luxol Fast Blue for myelin, DAPI for nuclei, and Alizarin Red for calcification assessment.

### 2.11 RT‐qPCR Gene Expression

NGCs, along with the regenerating neural tissues within them, were harvested at 1, 4, 8, 12, and 16 weeks post‐implantation. Samples (*n* = 3 biological replicates per time point) were immediately snap‐frozen at −80°C to preserve RNA integrity. Total RNA was extracted from the harvested tissues using the FavorPrep Blood/Cultured Cell Total RNA Mini Kit. Initially, tissues were pulverized and homogenized in liquid nitrogen. Subsequently, RNA was purified using silica columns and sequential ethanol washes, followed by elution in RNase‐free water. RNA concentration, purity, and integrity were confirmed by spectrophotometry and gel electrophoresis.

First‐strand cDNA was synthesized from total RNA using the Yekta Tajhiz cDNA Synthesis Kit with random hexamer primers. The reverse transcription reaction was carried out at 37°C for 60 min and terminated at 70°C.

Quantitative real‐time PCR (qPCR) was performed using YTA‐SYBR Green qPCR Master Mix 2X (*n* = 3 technical replicates for each biological replicate). Specific primers were designed for rat COL1A1, fibronectin 1 (FN1), tenascin C, and LAMB2. GAPDH was used as the internal reference gene for normalization (Table 1). Thermal cycling conditions included an initial denaturation at 95°C, followed by 40 cycles of denaturation, annealing, and extension. Melt curve analysis was conducted to confirm reaction specificity. Relative gene expression levels were calculated using the 2^‐ΔΔCt method and normalized to the 1‐week time point.

**TABLE 1:** Primer sequences for RT‐qPCR (Li et al. 2018, Rbia et al. 2020).

Primer	Sequence
GAPDH	Forward: 5'‐TACCAGGGCTGCCTTCTCTTG‐3'
	Reverse: 5'‐GGATCTCGCTCCTGGAAGATG‐3'
COL1A1	Forward: 5'‐AAGTCTCAAGATGGTGGCCG ‐3'
	Reverse: 5'‐TCGATCCAGTACTCTCCGCT ‐3'
Fibronectin1	Forward: 5'‐AGCCTGCCTCTTGCTCTTCCC ‐3'
	Reverse: 5'‐CGACTCTCCTCCCATCCACTCA ‐3'
LAMB2	Forward: 5'‐AGTACCCACACGGATGGAGTG ‐3'
	Reverse: 5'‐CTCGAGAACAGCCAGGTACA ‐3'
Tenascin C	Forward: 5'‐AAAGCAGCCACCCGCTATTAC ‐3'
	Reverse: 5'‐GATCTCCTCTGTCAAGACCTCAA ‐3'

### 2.12 Statistical Analysis

Data are expressed as mean ± SD. One‐way ANOVA with Tukey's post hoc test and repeated measures ANOVA were used for statistical analysis. Significance was set at *p* < 0.05. Graphs were generated using GraphPad Prism v10.5.0.

## 3 Results

### 3.1 Surface Morphology and Conduit Architecture

**FIGURE 2:**
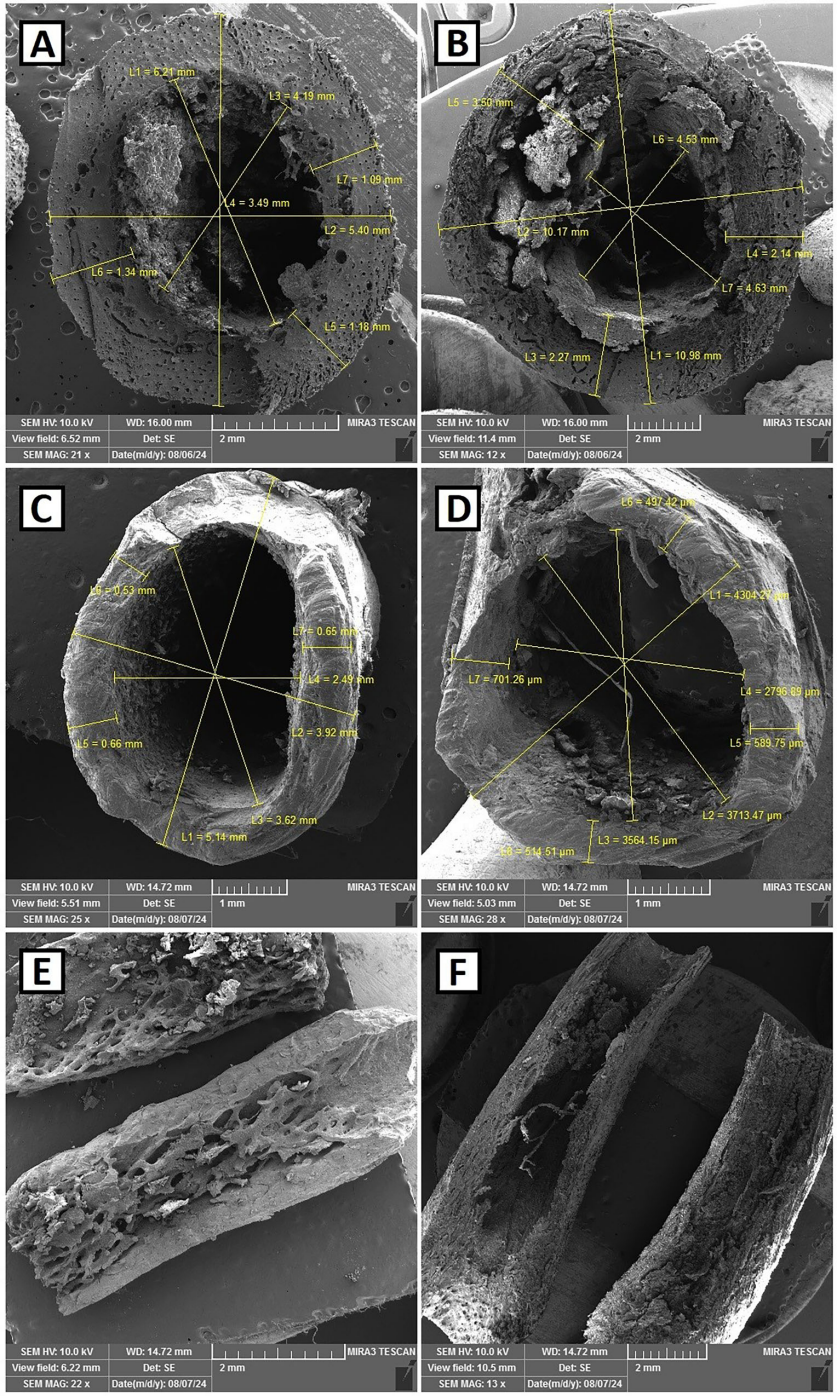
Field emission scanning electron microscopy (FESEM) transverse section pictures of (A) native and (B) demineralized rat femur and (C) native and (D) demineralized chicken ulna (A and C) showing the conduit wall thickness and lumen diameters as well as the longitudinal sections of (E) native and (F) demineralized bone conduits showing their porous architecture.

Field emission scanning electron microscopy (FESEM) of transverse and longitudinal sections of rat and chicken bone diaphyses revealed lumens ranging from oval to circular. Rat and chicken NGCs exhibited mean inner diameters of ≈4.5 and ≈2.8–3.6 mm, respectively; outer diameters of ≈10.5 and ≈4.3 mm, respectively; and wall thicknesses of ≈2.2 and ≈0.5 mm, respectively. Although wall thickness varied around each conduit's circumference, the luminal surfaces remained smooth and uniform, indicating consistent internal geometry despite irregular external dimensions. Longitudinal views displayed a trabecular‐like porous microarchitecture, confirming preservation of the native ECM framework (Figure 2).

### 3.2 Demineralization Efficacy

#### 3.2.1 Elemental Analysis

**FIGURE 3:**
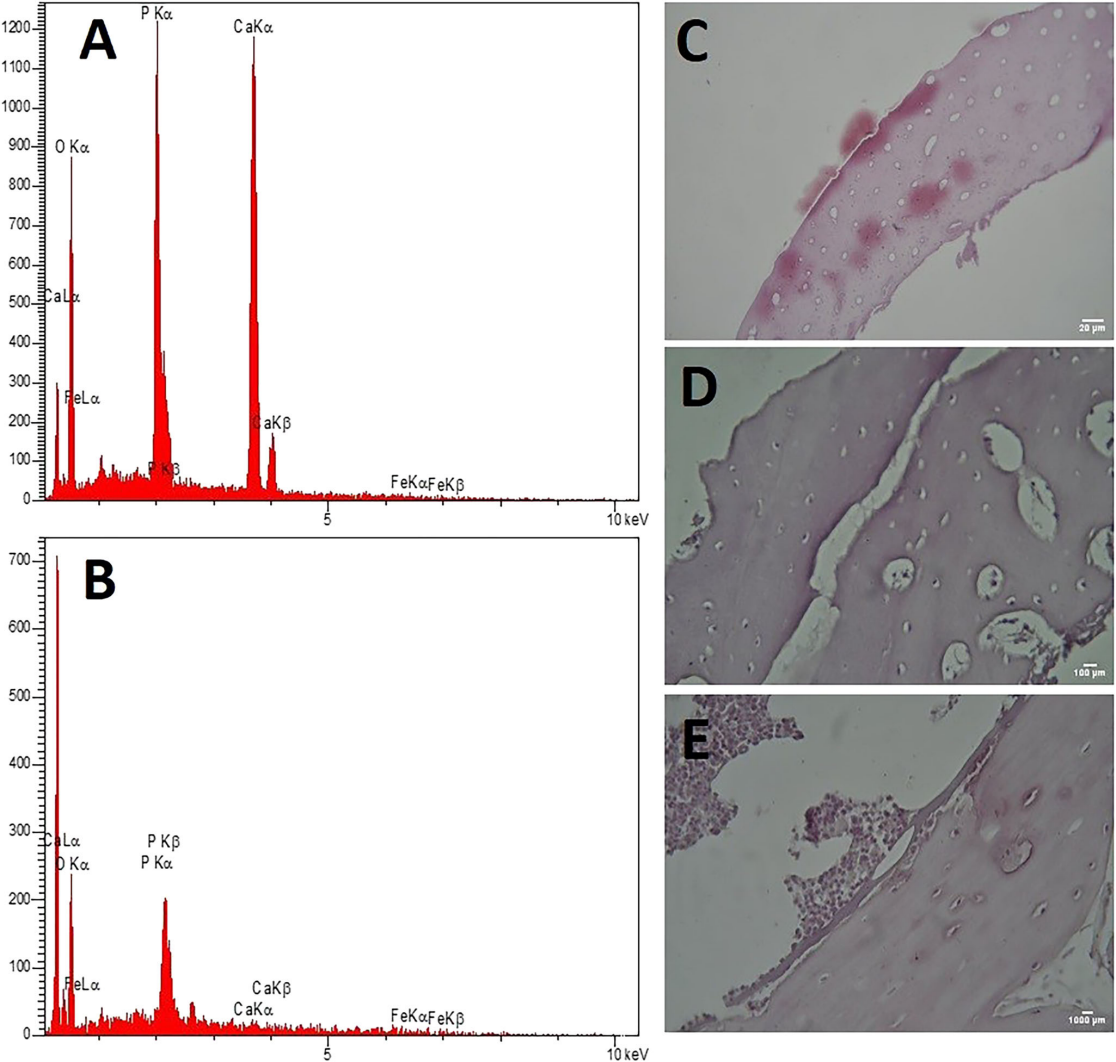
Energy dispersive X‐ray (EDX) spectrographs of bone samples (A) before and (B) after demineralization, showing phosphorus and calcium weight percentages. Alizarin Red staining of (C) red calcium deposits in an incompletely demineralized control; (D) a demineralized rat conduit; (E) demineralized chicken conduit—both lacking calcium staining.

Energy‐dispersive EDX quantified calcium content in native and demineralized bone samples. Native rat and chicken bones contained 29.40% and 32.71% Ca by weight, respectively, which decreased to 2.02% and 1.88% after demineralization (Figure 3A,B).

#### 3.2.2 Histological Validation

Alizarin Red staining corroborated the EDX findings: demineralized rat and chicken conduits showed no red calcium deposits, whereas partially demineralized controls retained distinct staining (Figure 3C–E). The combination of Alizarin Red imaging (visual confirmation) and EDX analysis (quantitative reduction) conclusively confirmed the efficacy of the demineralization process, validating these conduits for use as nerve guidance scaffolds.

### 3.3 Decellularization Verification

**FIGURE 4:**
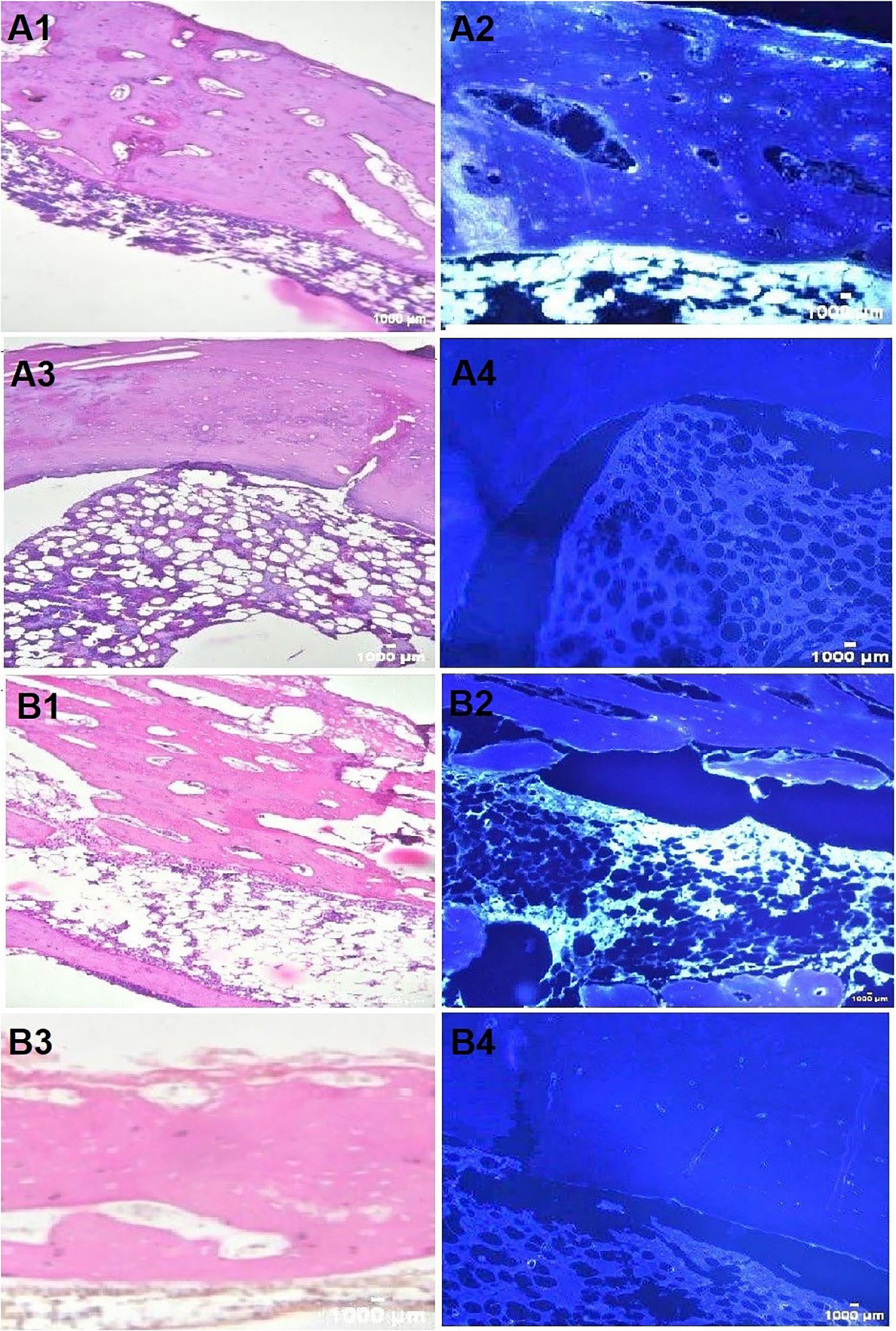
Representative images of H&E (left) and DAPI (right) staining in native and decellularized rat femur and chicken ulna. nuclei (dark purple in H&E; bright blue in DAPI), which are absent post‐decellularization.

Hematoxylin‐eosin and DAPI staining of rat femur and chicken ulna sections demonstrated complete removal of cellular nuclei in decellularized conduits, whereas native controls exhibited abundant nuclear staining (Figure 4). These results confirm the full decellularization of both bone‐derived NGCs.

### 3.4 Collagen Preservation

**FIGURE 5:**
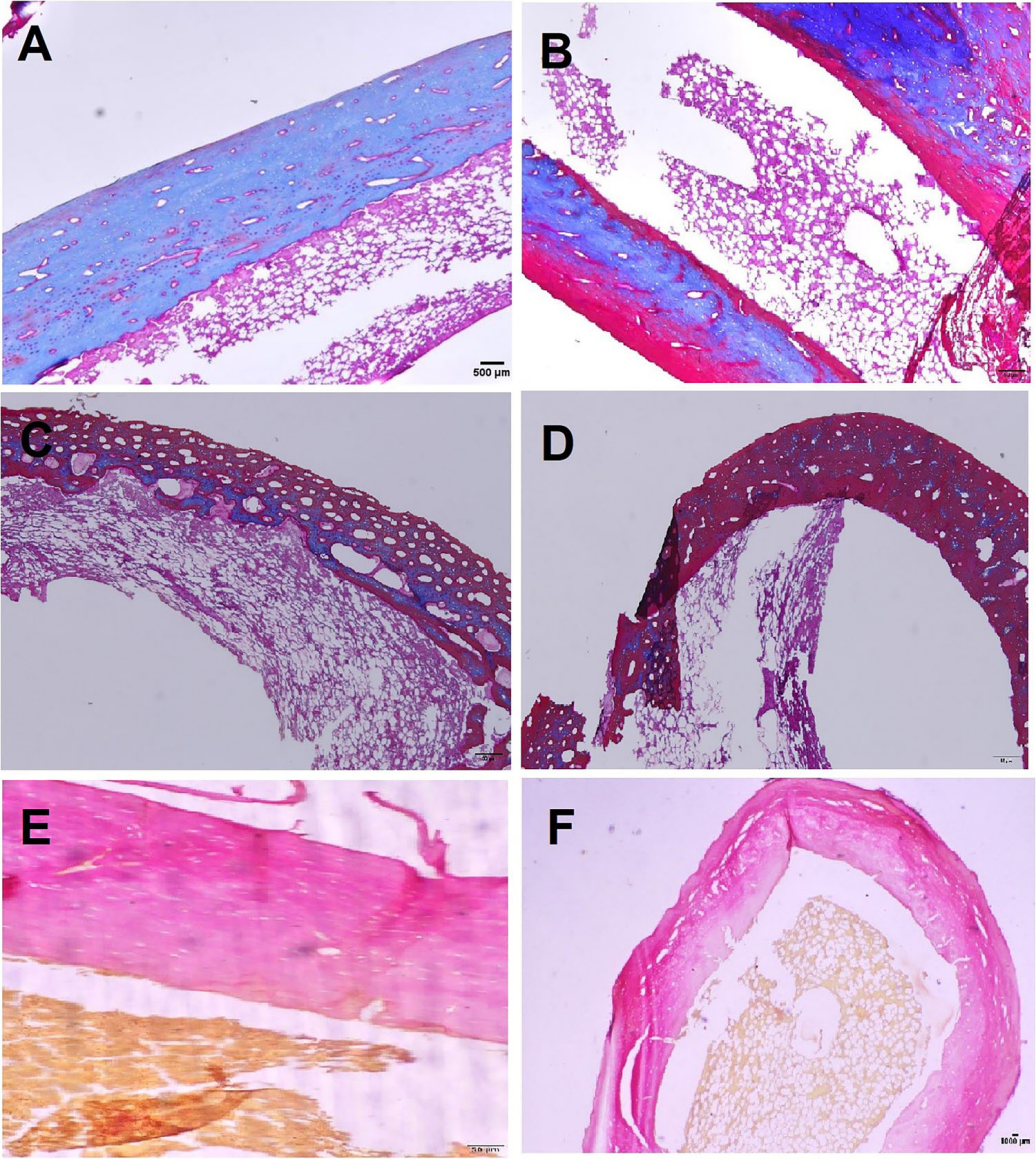
Collagen visualization in bone conduits using histological staining techniques. (A and C) Demineralized NGCs derived from rat and chick, respectively. (B and D) Demineralized and decellularized NGCs from rat and chick, respectively. Masson's trichrome staining reveals collagen fibers in blue‐green tones. (E and F) Picrofuchsin staining highlights collagen presence in rat and chick NGCs, respectively, with red coloration under polarized light microscopy.

Collagen content was assessed via Masson's trichrome and picrofuchsin staining (Figure 5). Masson's trichrome revealed blue–green collagen fibers in both demineralized (A and C) and demineralized–decellularized (B and D) rat and chicken conduits. Picrofuchsin staining further confirmed the presence of red‐stained collagen fibrils after demineralization and decellularization (E and F), indicating relative preservation of the collagen matrix within the conduits.

### 3.5 Mechanical Characterization

**FIGURE 6:**
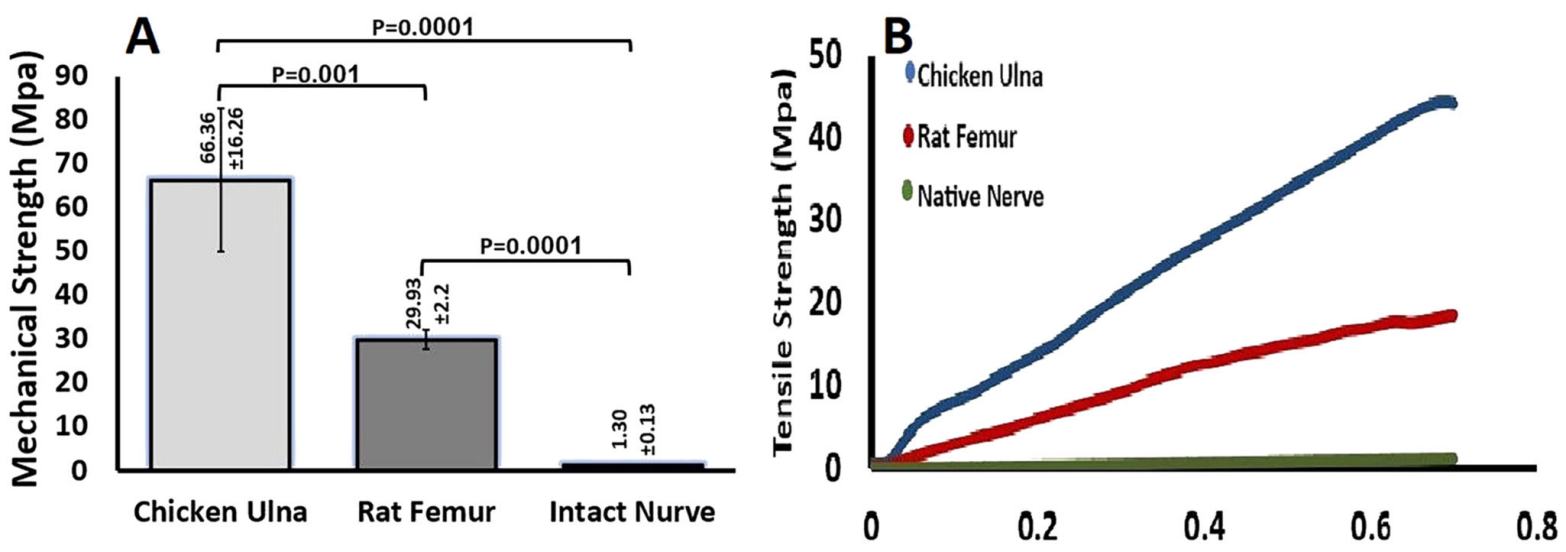
(A) Mean Young's modulus (MPa) (one‐way ANOVA); (B) Representative force–strain curves for experimental groups, with *R* ^2^ values.

Uniaxial tensile testing of decellularized bone conduits and native nerve grafts produced linear force–strain relationships in the elastic region, consistent with Hooke's law. Young's modulus was calculated from the slope of this region, and the coefficient of determination (*R* ^2^) confirmed data fit. Chicken ulna conduits exhibited the highest Young's modulus (66.36 ± 16.26 MPa), followed by rat femur conduits (39.93 ± 2.2 MPa) (Figure 6A–B). Ultimate tensile strength comparisons similarly showed superior values for chicken conduits (Figure 6C).

### 3.6 Electrical Conductivity

Wet conduits from rat femur and chicken ulna exhibited conductivities of 1.01 × 10^−^^5^ S/cm and 1.11 × 10^−^^5^ S/cm, respectively, whereas their dry counterparts measured 4.77 × 10^−^^10^ S/cm and 1.21 × 10^−^^9^ S/cm (Table 2). Thus, hydration increased conductivity by approximately four orders of magnitude, underscoring the potential role of ionic pathways in the regenerating microenvironment.

**TABLE 2:** Electrical conductivity of bone conduits.

Conduit type	Dry *σ* (S/cm)	Hydrated *σ* (S/cm)	Fold increase
Rat femur diaphysis	4.77 × 10^−^^10^	1.01 × 10^−^^5^	∼2.1 × 10^4^
Chicken ulna	1.21 × 10^−^^9^	1.11 × 10^−^^5^	∼9.2 × 10^3^

### 3.7 Cytocompatibility (MTT Assay)

**FIGURE 7:**
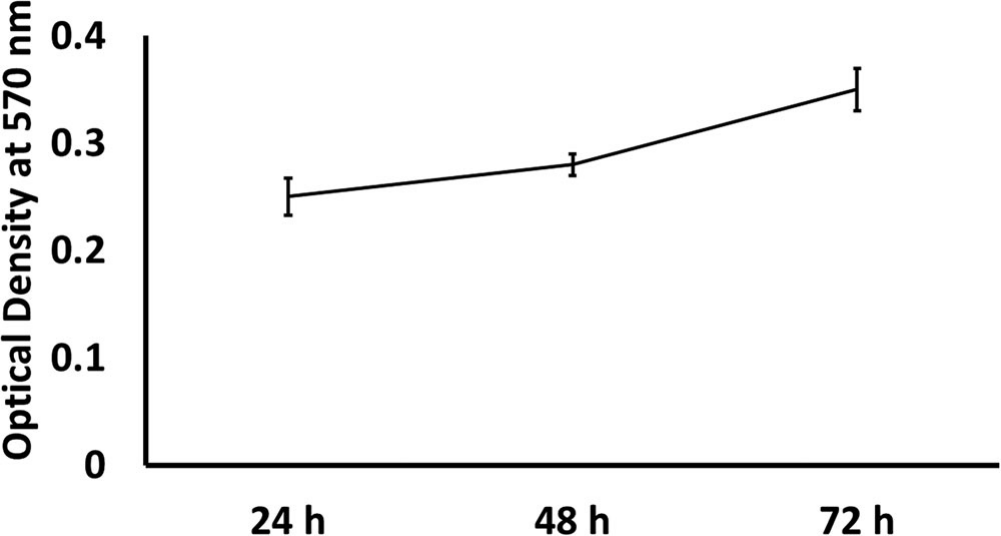
MTT assay showing an upward trend for cell activity during a 72 h time period, indicating the non‐toxicity of the decellularized bone conduit.

MTT assays measured the metabolic activity (at 570 nm OD) of rat mesenchymal stem cells cultured on decellularized bone conduit at 24, 48, and 72 h. These results indicate the non‐toxicity of the demineralized/decellularized bone conduit, thus confirming the removal of chemicals used during the demineralization and decellularization processes (Figure 7).

### 3.8 Macroscopic Assessment of Conduit Persistence and Nerve Appearance

**FIGURE 8:**
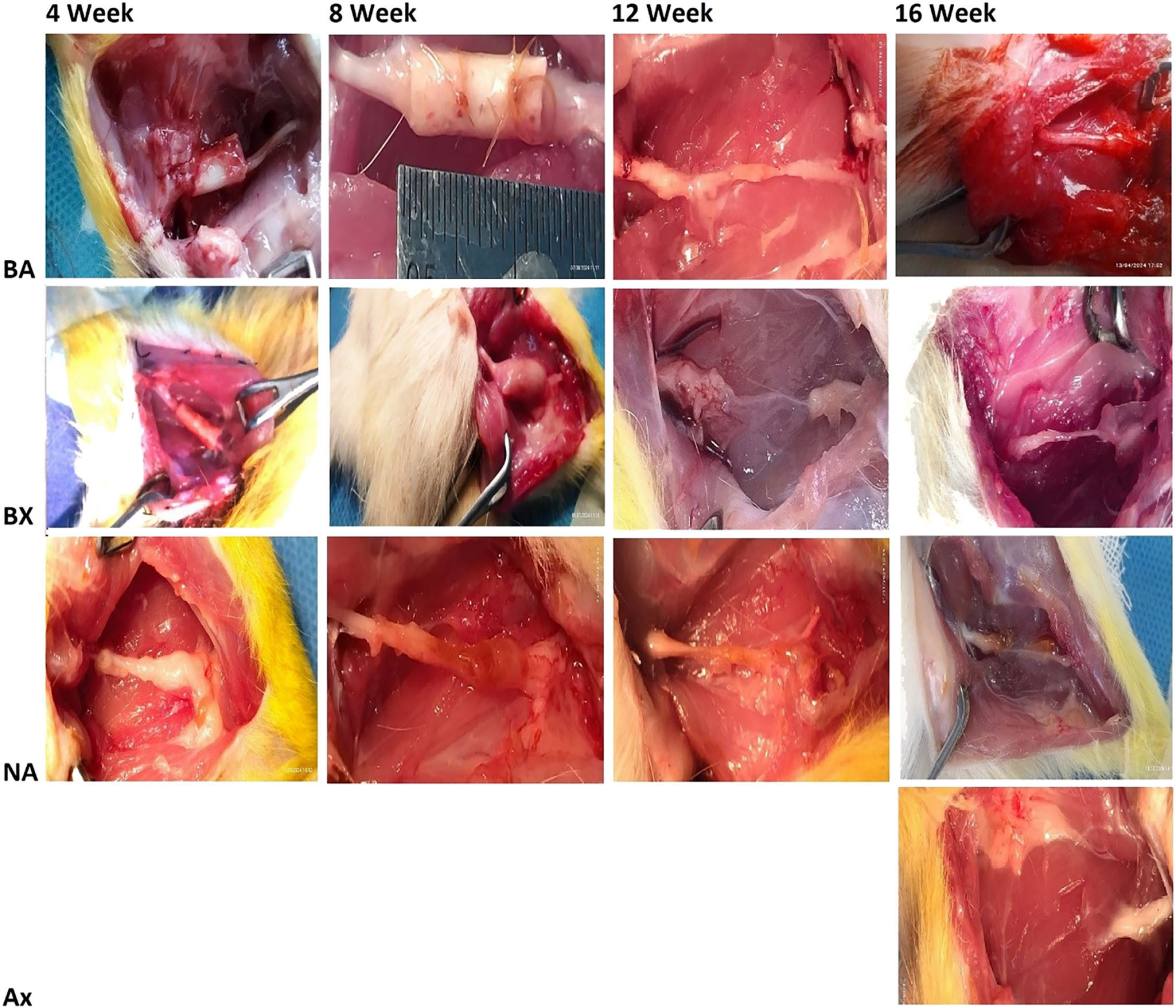
Digital images of the repair site at weeks 4, 8, 12, and 16 in Rat bone allograft (BA) conduit, Chicken bone xenograft (BX) conduit, Nerve allograft (NA), and Axotomy (Ax) groups. Bone conduits are clearly visible through week 8.

Gross inspection of the repair site post‐implantation (Figure 8) revealed that bone conduits remained intact and visible at 4 and 8 weeks. By 12 and 16 weeks, conduits appeared fully resorbed and were replaced by regenerating nerve tissue.

### 3.9 Motor Function Recovery (SFI)

**FIGURE 9:**
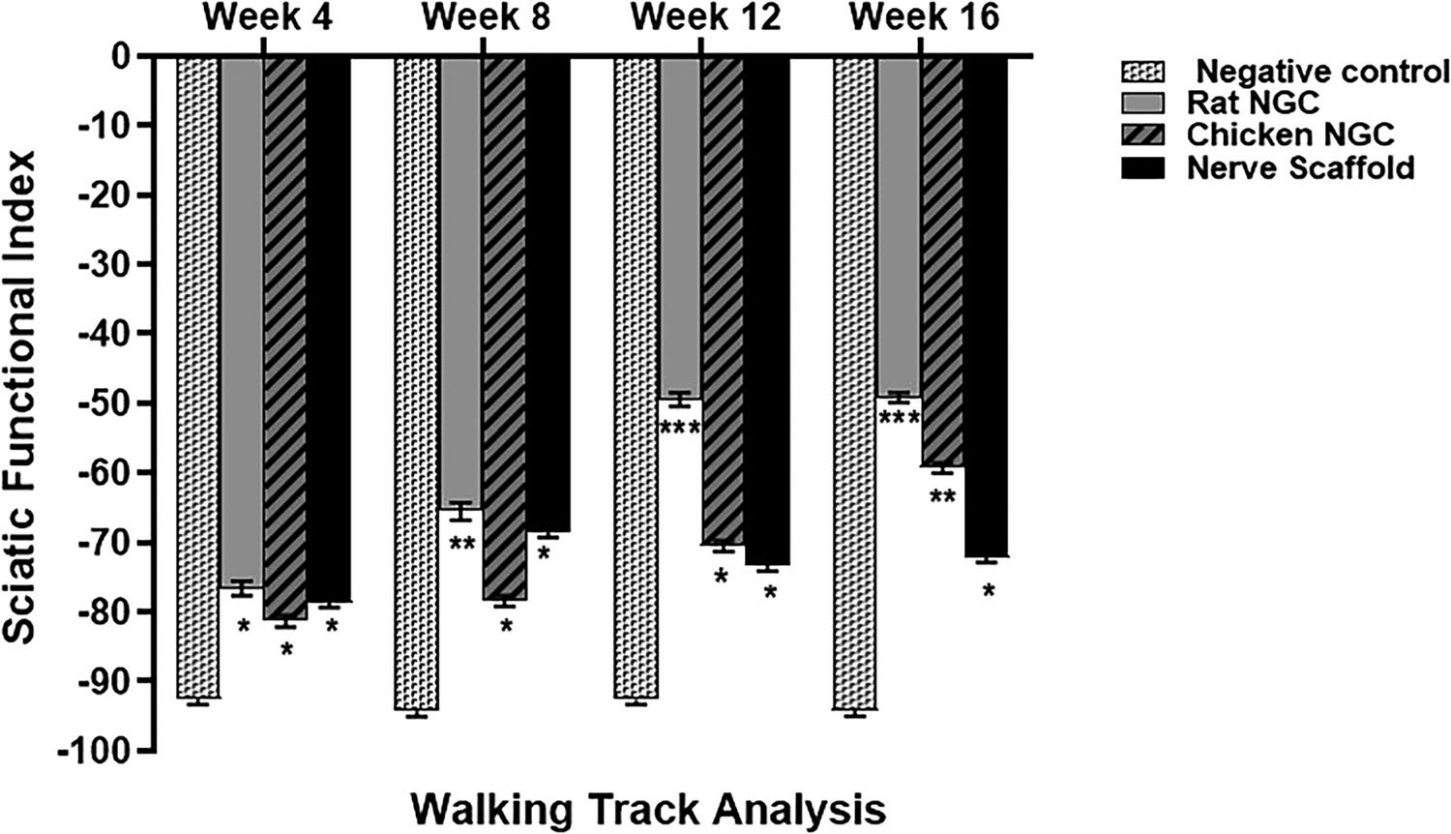
SFI values over 4, 8, 12, and 16 weeks for each group (mean ± SD) (repeated measure ANOVA; *p < 0.05, **p < 0.01, ***p < 0.001 versus Axotomy).

The SFI ranges from 0 (normal) to –100 (complete dysfunction). Axotomy rats remained near –100 throughout the study period. Compared with the Axotomy group, the Rat NGC group showed significantly greater improvement in sciatic nerve function at all time points. The Chicken NGC and Nerve Scaffold groups showed moderate improvement (Figure 9).

### 3.10 Sensory Function Recovery (Hot‐Plate Test)

**FIGURE 10:**
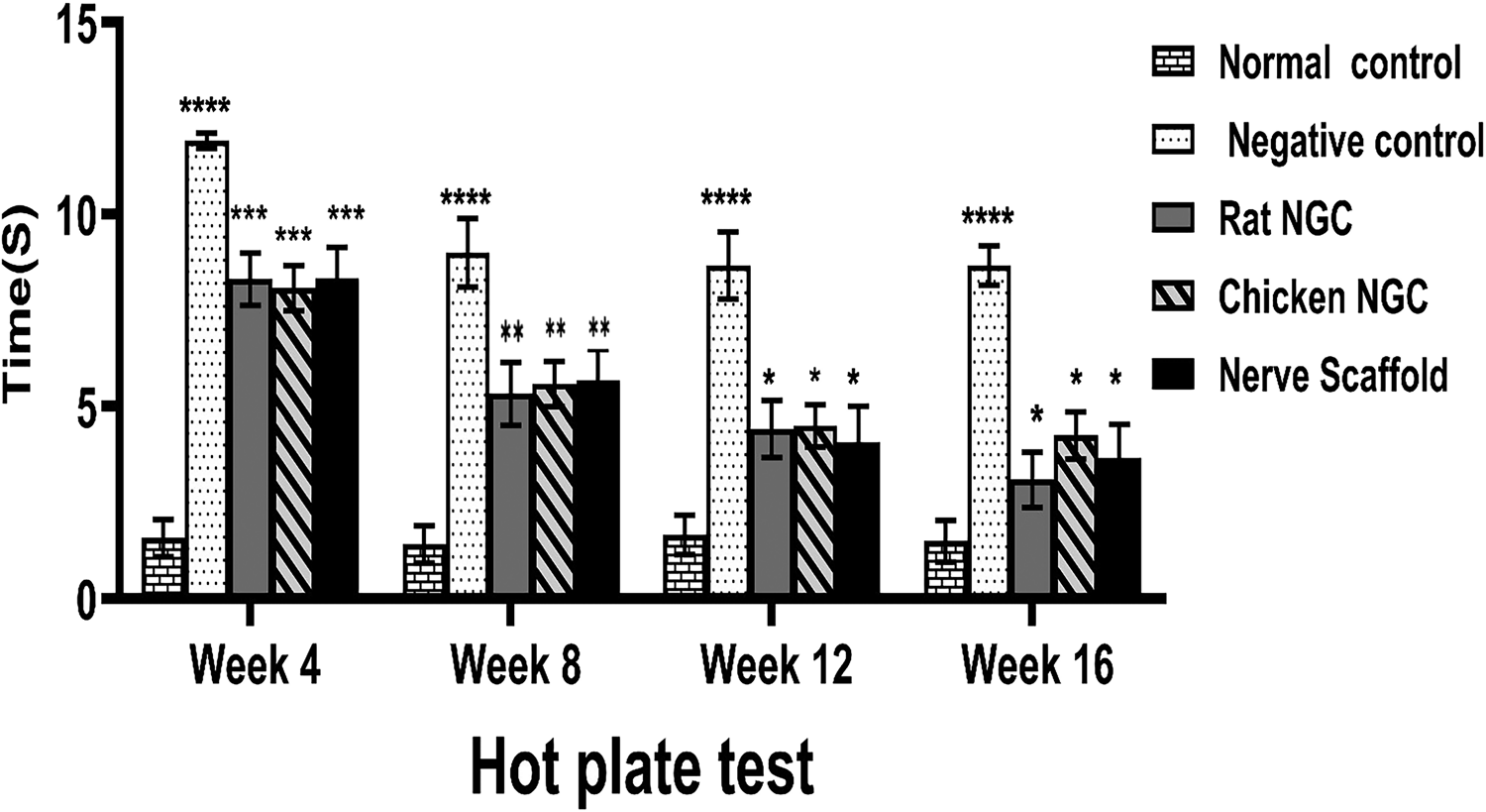
Hot‐plate withdrawal latency at 4, 8, 12, and 16 weeks (mean ± SD). (repeated measure ANOVA; asterisks denote significant differences versus Sham: *p < 0.05, **p < 0.01, ***p < 0.001, ****p < 0.0001).

Thermal nociception latency on a 55°C hot plate was significantly prolonged in the axotomy group compared to the Sham group at all time points (****p < 0.0001). All treatment groups demonstrated enhanced sensory recovery compared to the Axotomy group, but none returned to Sham levels by 16 weeks (*p < 0.05) (Figure 10).

### 3.11 Gastrocnemius Muscle Mass Ratio

**FIGURE 11:**
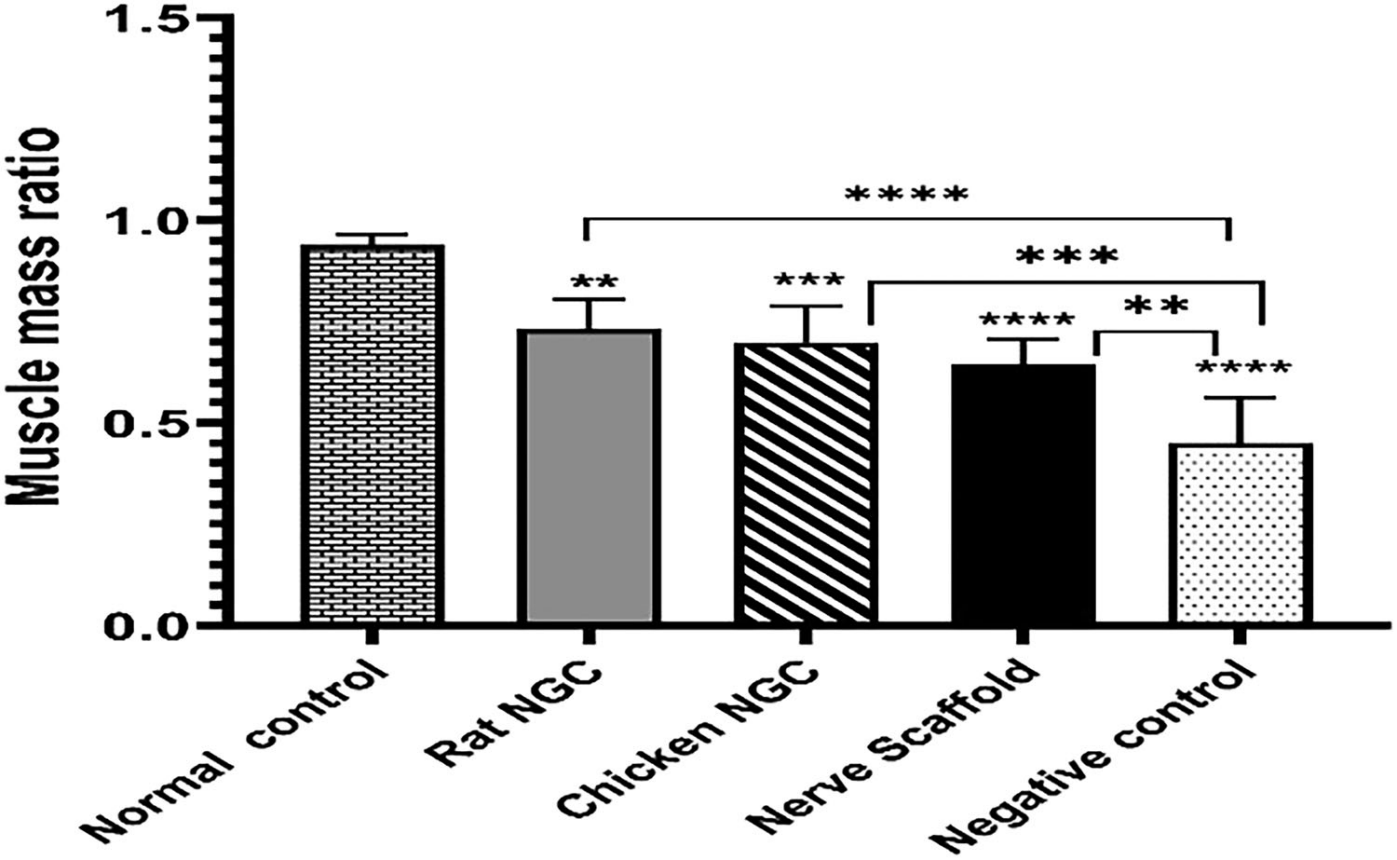
Gastrocnemius mass ratio at 16 weeks (mean ± SD). (One way ANOVA; **p < 0.01, ***p < 0.001, ****p < 0.0001 versus normal control and negative control.

**FIGURE 12:**
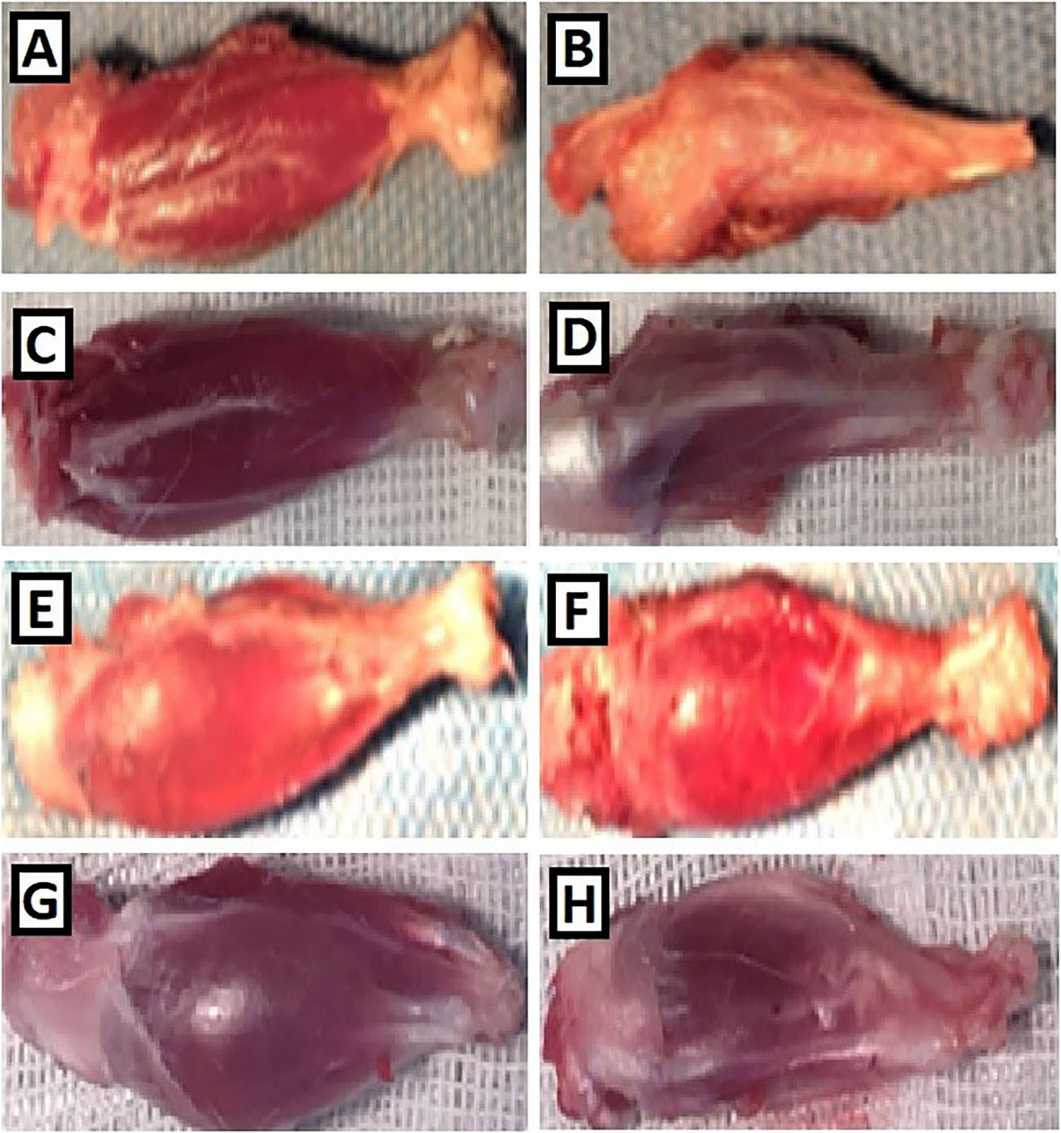
Rat Gastrocnemius muscle in (A) intact (control) and (B) axotomized; (C) intact (control) and (D) axotomized with a nerve graft; (E) intact (control) and (F) axotomized with a rat femur conduit graft; and (G) intact (control) and (H) axotomized with a chicken ulna conduit graft.

At week 16, the muscle mass ratio (operated/contralateral) was markedly reduced in the Axotomy group (****p < 0.0001 vs. Sham). The Rat and Chicken conduit groups partially preserved muscle mass, with both showing significantly greater ratios than the Axotomy group but below those of the Sham group. No significant difference was observed between the two bone conduit groups (Figures 11 and 12).

### 3.12 Histological Assessments

#### 3.12.1 H&E and Picrofuchsin Staining

**FIGURE 13:**
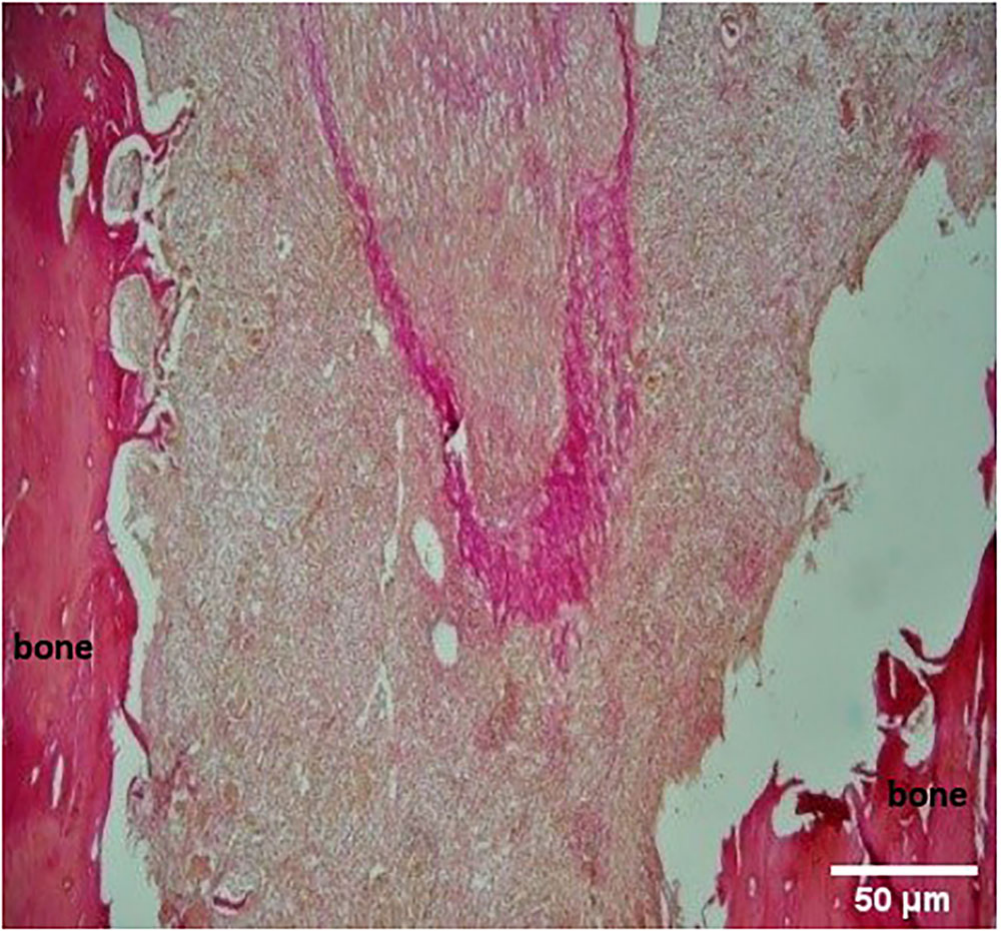
Picrofuchsin–stained longitudinal section at week 4, showing nerve fiber advance inside rat bone conduit. The epineurium (pink collagen) and immune cells are visible.

**FIGURE 14:**
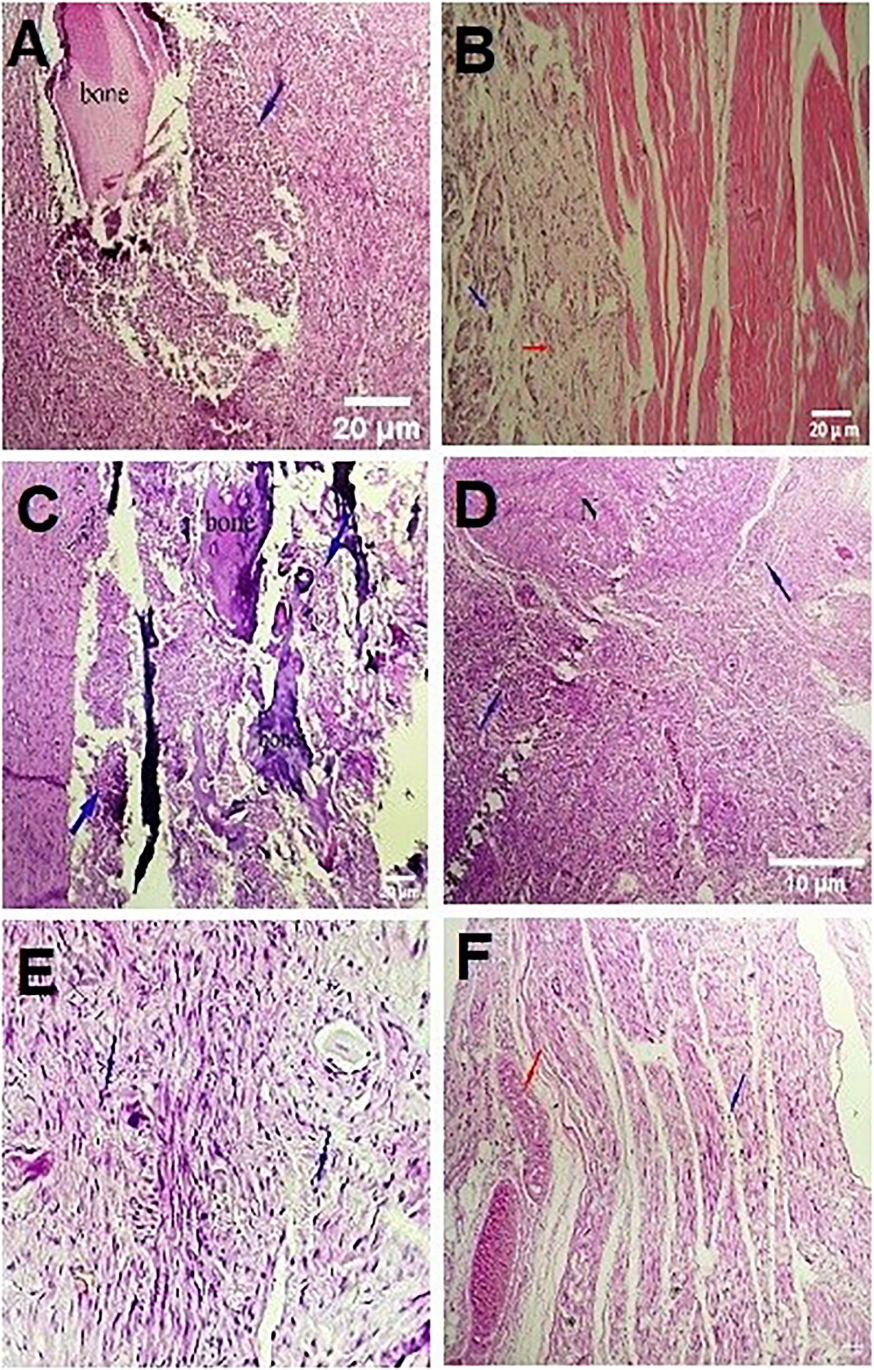
Week 4 histology by H&E: (A and B) rat allograft conduit and (C and D) chicken xenograft, showing adhered cells on bone surface, regenerating sciatic nerve fibers (N), dense inflammatory infiltrate (blue arrows), and blood vessels (red arrows). (E and F) Rat nerve allograft group with inflammatory cells and multinucleated giant cells (blue arrows).

At 4 weeks post‐implantation, H&E and Picrofuchsin staining of the nerve‐allograft and bone conduit groups revealed early axonal infiltration and initial nerve fiber formation within the conduits (Figure 13). A robust inflammatory response was evident around the graft, with infiltrating immune cells (indicated by blue arrows). Furthermore, cell adhesion to the conduit walls was observed, underscoring the scaffold's capacity to sequester and present pro‐regenerative cellular and molecular cues. Early neovascularization was also noted, with capillary‐like structures formed by endothelial cells throughout the regenerating tissue (indicated by red arrows) (Figure 14A–D). Similar inflammatory and vascular responses occurred around the decellularized NA (Figure 14E–F). By 8 weeks, the inflammatory infiltrate had markedly subsided. By 12 weeks, the bone conduits were largely degraded and replaced by regenerating nerve tissue.

#### 3.12.2 Luxol Fast Blue Staining (LFB)

**FIGURE 15:**
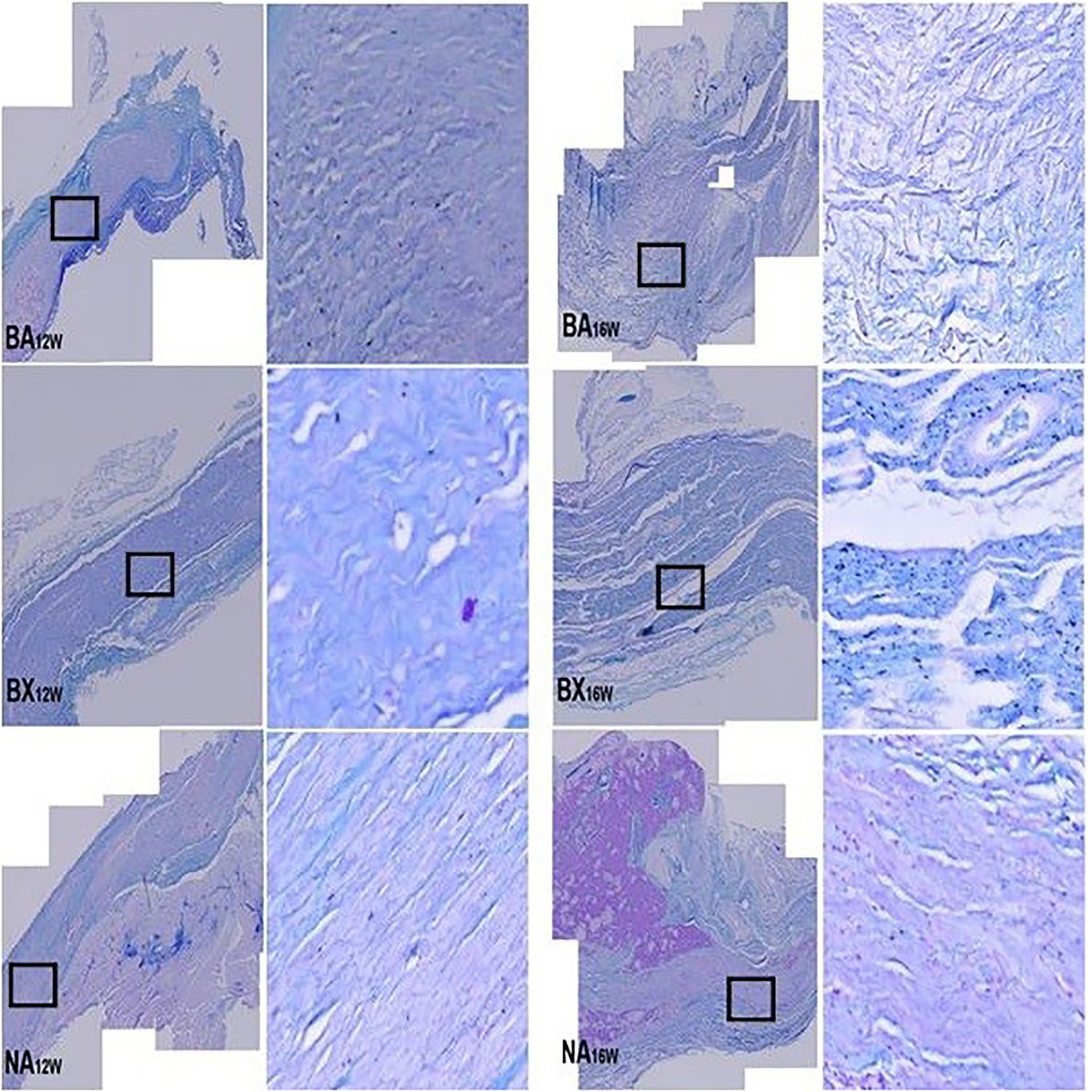
Luxol Fast Blue staining at weeks 12 and 16. Emerging myelin layers (blue to blue‐green) reflect advancing remyelination. (4x; assembled by Microsoft ICE v2.0.3.0.).

LFB specifically stains myelin sheaths blue to blue‐green, while neuronal cell bodies and unmyelinated axons remain unstained. At 4 and 8 weeks, myelin was not yet prominent, consistent with early axonal regeneration typically lacking a mature myelin layer. By 12 and 16 weeks, distinct myelin layers appeared (darker blue in areas of higher myelin density), indicating progressive re‐myelination (Figure 15). Images were captured at 4× magnification and assembled using Microsoft ICE 2.0.3.0 software.

#### 3.12.3 Masson's Trichrome Staining

**FIGURE 16:**
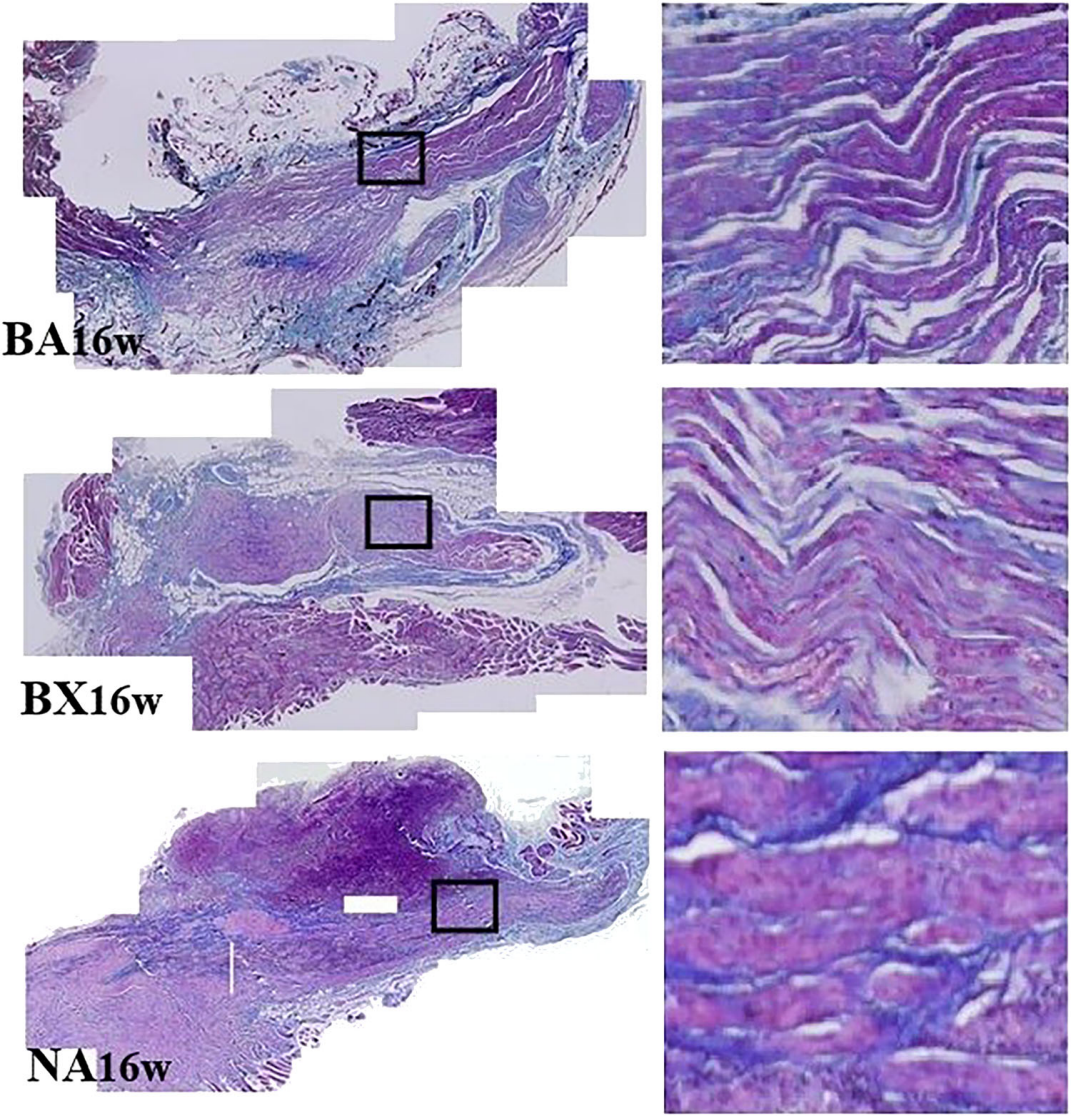
Masson's Trichrome at week 16 showing collagen within endoneurium, perineurium, and epineurium (blue/green) (4×; assembled by ICE v2.0.3.0.).

Masson's trichrome staining highlights collagen in endoneurium, perineurium, and epineurium as blue or green. At 16 weeks, the regenerating nerve demonstrated well‐organized connective tissue layers surrounding regenerated fibers (Figure 16).

### 3.13 Quantitative Evaluation of ECM Gene Expression

**FIGURE 17:**
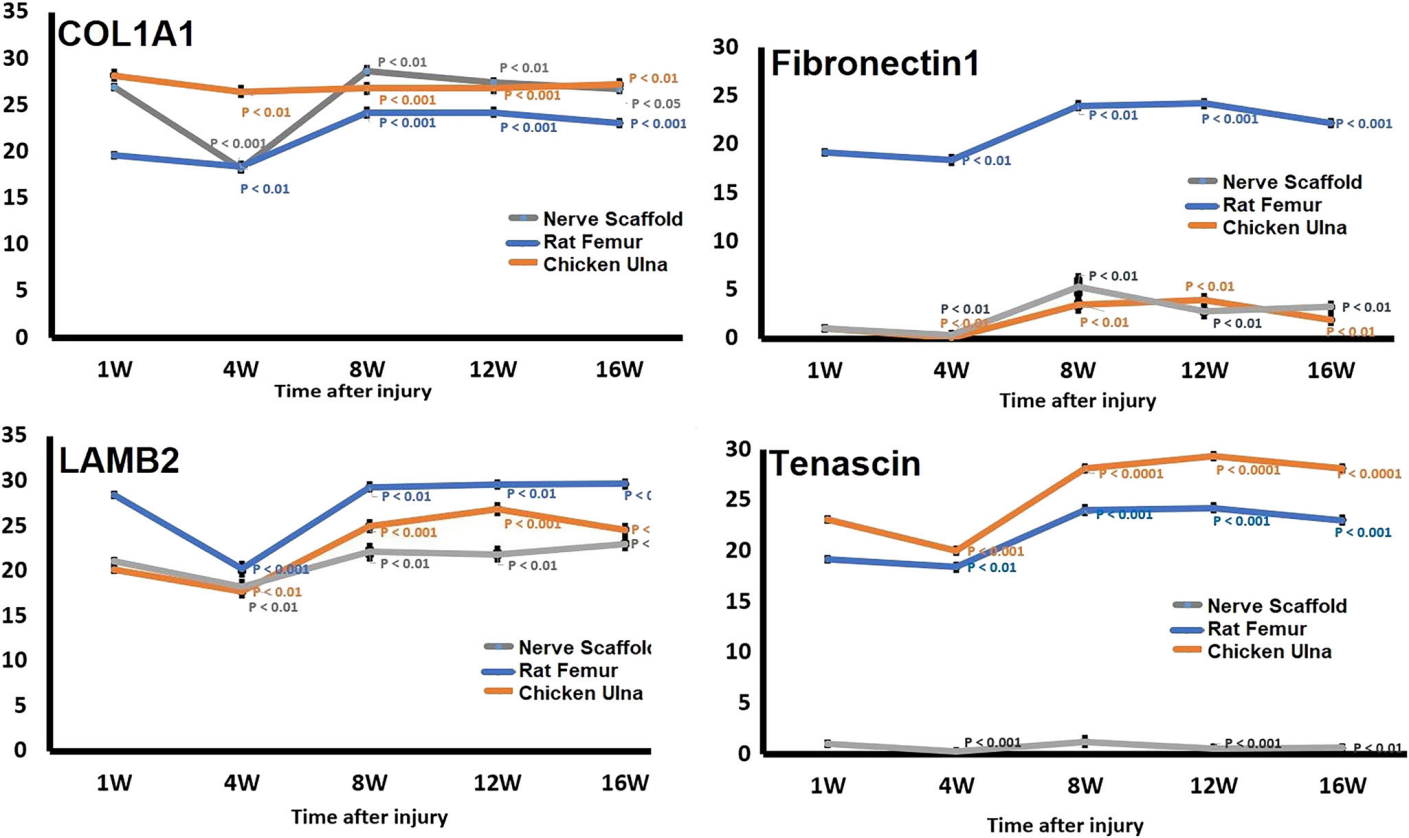
Expression profiles of COL1A1, FN1, LAMB2, and Tenascin C genes in three construct types: decellularized nerve, rat femur bone conduit, and chicken ulna bone conduit over a 16‐week period following surgery. The chicken bone conduit showed the highest expression of COL1A1 and Tenascin C at week 1, indicating early ECM response. The rat femur conduit exhibited peak FN1 and LAMB2 expression at week 8, reflecting active ECM remodeling and basement membrane reconstruction. The nerve scaffold showed a delayed FN1 peak at week 12, suggesting a slower regenerative trajectory. Expression trends corresponded to distinct phases of nerve repair, including initial ECM activation, cellular proliferation, and matrix stabilization. Statistical significance was determined using ANOVA followed by post hoc analysis. Error bars represent standard deviation (SD); (*n* = 3).

This study investigated the expression profiles of ECM‐related genes across three construct types, decellularized nerve tissue, rat femoral conduit, and chicken ulna conduit, during a 16‐week period following sciatic nerve surgery. Temporal expression patterns were observed for four key genes: COL1A1, FN1, LAMB2, and Tenascin‐C, each corresponding to distinct phases of neural tissue regeneration (Figure 17).

- COL1A1 (type I collagen): At week 1, the chicken bone scaffold exhibited the highest expression level of COL1A1. By week 4, a decline was observed in all scaffold groups. From week 4 to 8, expression levels gradually increased, peaking at week 8. Thereafter, a plateau persisted until week 16.
- FN1 (fibronectin): The rat femoral bone scaffold showed elevated FN1 expression at week 8. The nerve scaffold exhibited a delayed peak at week 12. The chicken scaffold maintained moderate and relatively stable FN1 expression.
- LAMB2 (laminin beta‐2): A high LAMB2 expression was seen in the rat femoral bone conduit. The nerve scaffold displayed relatively low levels of laminin beta‐2 expression. The chicken scaffold exhibited intermediate expression.
- Tenascin‐C: Tenascin‐C expression peaked at week 1 in the chicken scaffold, followed by a decline at week 4 across all groups. A second increase was detected at week 8, followed by a plateau from week 12 onward.

## 4 Discussion

One of the major challenges in peripheral nerve repair is achieving effective reconnection between the severed nerve ends, particularly in cases involving large gaps where direct suturing is not feasible and cannot anastomose the nerve. In this study, to evaluate the efficacy of acellular and demineralized bone conduits, scaffolds were prepared by removing cellular components and minerals, thereby creating a favorable microenvironment for cellular guidance. SEM images (Figure 2) revealed that these scaffolds possessed aligned and organized structures, functioning as guiding channels for neural cell growth.

A notable design consideration is the dimensional mismatch between the conduit lumens (≈4.5 mm for rat femur and ≈2.8–3.6 mm for chicken ulna) and the native rat sciatic nerve (≈1.5–2 mm diameter). While this oversizing was necessitated by the anatomical constraints of the donor bones and facilitated surgical handling and suturing, it may have implications for axonal guidance efficiency. Larger lumens reduce the probability of direct contact between regenerating axons and the luminal wall, potentially delaying the ‘contact guidance’ mechanism that typically directs axonal extension along aligned ECM fibers (Yan et al. 2022). However, the porous trabecular architecture observed in our SEM analysis (Figure 2) may partially compensate for this mismatch by providing a three‐dimensional scaffold network that supports axonal infiltration even without direct wall contact. Previous studies have shown that oversized conduits can still support functional regeneration if they contain intraluminal fillers or porous structures that guide axonal growth (Gong et al. 2022; Millesi et al. 2023). The favorable SFI and histological outcomes observed in our study (Figures 9 and 13–15) suggest that the mismatch did not critically impair regeneration, possibly due to the preserved collagen matrix and natural porosity of bone‐derived scaffolds. Nevertheless, future studies should explore optimizing conduit diameter or incorporating intraluminal guidance structures to minimize the lumen‐to‐nerve ratio and enhance directional axonal growth.

Following implantation of these conduits into a 10‐mm gap in the rat sciatic nerve, histological, electrophysiological, and functional assessments confirmed successful tissue integration, restoration of neural signal transmission, and significant recovery of limb function.

Comparable findings have been reported in previous studies. X. Li et al. (2022) conducted a systematic review on collagen‐based scaffolds for peripheral nerve repair and emphasized their biocompatibility, structural integrity, and ability to support axonal regeneration. Mao et al. (2023) demonstrated that ECM and PLA‐PCL based conduits promoted axonal migration and tissue‐level reconnection in a rat model. Similarly, Nazeri et al. (2022) used PCL‐based microchannel conduits and showed, via SEM and histology, that these structures facilitated directional cell migration. Compared to these studies, the present work offers a multi‐layered evaluation combining SEM, histology, Luxol LFB, and microscopic analysis along with a quantitative estimation of the number of cells required to bridge the conduit, highlighting the precision and depth of the experimental design.

Despite promising results, the current study design had certain limitations. Although the rat model offers experimental advantages, its translational relevance to human physiology remains limited. Therefore, future studies should consider larger animal models. Additionally, the absence of multiple control groups and detailed molecular analyses may influence the interpretation of outcomes. As an alternative, chicken bone was used due to its low cost and ease of breeding. Nevertheless, this study provides valuable preliminary insights into the performance of bone‐derived conduits.

The first objective of the study was to assess the potential for tissue‐level reconnection between the proximal and distal nerve stumps. Following decellularization and demineralization, the bone conduits exhibited a collagenous, cell‐free, and calcium‐depleted structure in SEM and histological images (Figures 2, 3, 4, 5). These features are expected to reduce immunogenic responses and calcification, while providing a suitable substrate for neural plasticity. Light microscopy (Figures 13 and 14) clearly illustrated the internal architecture of the scaffold, the positioning of neural tissue within it, and the progression of regeneration. Luxol LFB (Figure 15) further demonstrated that the proximal and distal nerve ends had migrated toward each other, establishing tissue continuity and initiating axonal myelination.

Considering the 10‐mm gap (10,000 µm) and the average size of rat sciatic nerve cells (5–10 µm), it can be estimated that around 1000–2000 cells must bridge the conduit to achieve effective reconnection. This highlights the complexity and critical importance of precise conduit design in facilitating cellular guidance.

The native sciatic nerve is mechanically compliant. Therefore, provided that the gap length after injury and transection is not too long, the proximal and distal stumps can be approximated and essentially grafted together using sutures.

In this study, where a long gap (10 mm) was considered, the mechanical compliance of the sciatic nerve allowed the proximal and distal stumps to be pulled into the processed bone conduit using suture threads. In this situation, it is only necessary for the processed bone conduit to possess sufficient structural integrity to function as a channel and to allow suture knots to be tied over it without causing its damage. Moreover, despite the significant difference in stiffness between the processed bone conduits derived from chick ulna and rat femur, both had adequate strength to serve as effective conduits.

The mechanical characterization revealed that both bone‐derived conduits exhibited Young's modulus values (39.93 ± 2.2 MPa for rat femur and 66.36 ± 16.26 MPa for chicken ulna) that are substantially higher than native rat sciatic nerve tissue, which typically ranges from 0.5 to 5 MPa in the elastic modulus (Wang et al. 2024; Petit et al. 2024). This stiffness mismatch warrants careful consideration of potential biological consequences. While we emphasize that the mechanical properties were sufficient for suturing and structural integrity during implantation, the supraphysiological stiffness may influence cellular behavior through mechanotransductive pathways. Schwann cells and neurons are known to respond to substrate stiffness, with excessively rigid scaffolds potentially promoting fibroblast proliferation and collagen deposition, leading to intraneural fibrosis and impaired axonal regeneration (Montgomery et al. 2025). Additionally, stiffness mismatch at the nerve‐conduit interface can concentrate mechanical stress, potentially triggering chronic inflammatory responses or foreign body reactions (Gori et al. 2021). Despite these theoretical concerns, our histological findings at 16 weeks (Figures 13, 14, 15, 16) did not reveal overt fibrosis or persistent inflammation beyond the expected early remodeling phase. The progressive conduit resorption observed between weeks 8 and 12 (Figure 8) likely mitigated long‐term mechanical mismatch, as the degrading scaffold gradually transferred mechanical load to the regenerating tissue. This temporal degradation profile may represent an advantageous feature of bone‐derived conduits compared to non‐degradable synthetic alternatives. However, future studies should directly investigate the impact of stiffness mismatch on Schwann cell phenotype, fibroblast activation, and long‐term functional outcomes, possibly through the use of softer decellularized scaffolds or composite materials that more closely mimic native nerve mechanics (Wang et al. 2024; Carvalho et al. 2021).

The second objective was to evaluate the establishment of electrophysiological connectivity between the nerve ends. SFI and thermal sensitivity assessments (Figures 9 and 10) suggested that neural signal transmission was likely restored and sensorimotor function improved following conduit implantation. However, as direct electrophysiological measurements (e.g., nerve conduction velocity or EMG) were not performed, conclusions regarding definitive restoration of nerve conductivity remain indirect and should be interpreted with caution. As a limitation, direct nerve conduction velocity or EMC measurements, which could confirm the passage of electrical signals through the regenerated nerve, were not done in this study; however, the continuity between the distal and proximal ends shown in the histology, coupled with favorable sensory and motor test outcomes, suggests interpolation of results and confirms that nerve conductivity was indeed re‐established.

Similar outcomes were reported by Taifebagerlu et al. (2015), who used silicone conduits and assessed EMG and SFI parameters. Mao et al. (2023) demonstrated restored signal conduction following the employing of ECM‐coated conduits. Zheng et al. (2021) utilized nanofiber scaffolds and confirmed functional recovery through behavioral tests. Compared to these studies, the present work integrates three distinct functional assays, offering a comprehensive evaluation of neural reconnection.

The third objective was to assess the impact of nerve regeneration on limb function recovery. Imaging of the gastrocnemius muscle (Figure 11) revealed reduced muscle atrophy and preserved muscle volume in the conduit‐treated group. These findings suggest that the reestablished neural connection successfully prevented secondary muscle degeneration and maintained motor function. Additionally, behavioral observations several weeks post‐surgery showed substantial recovery of locomotor ability, further confirming functional restoration of the affected limb.

Comparable studies include Yu et al. (2011), who evaluated electrophysiological and muscle reinnervation using collagen/poly(ε‐caprolactone) nerve conduits; Ahmadi et al. (2025), who used PCL/CNT/EGCG nerve conduits and assessed muscle atrophy; and Hu et al. (2016), who demonstrated improved reinnervation with cryopolymerized gelatinmethacryloyl (cryoGelMA) gel scaffolds through functional and histological assessments. The present study offers enhanced resolution through imaging and long‐term behavioral analysis, underscoring the functional relevance of the conduit design.

At the molecular level, the expression of key ECM genes such as collagen1a1, laminin B2, fibronectin, and tenascin‐C plays a pivotal role in nerve regeneration.

Collagen I provides mechanical support and adhesion sites for neural cells. In our study, the highest expression level of COL1A1 at week 1 in the chicken bone scaffold is likely due to its larger contact area and superior cell adhesion potential. The gradual increase from week 4 to 8 aligns with cellular proliferation and active collagen matrix production. And finally, the persisting plateau until week 16 is likely reflecting scaffold saturation and reduced cellular growth rate.

Fibronectin contributes to cell adhesion, migration, and early tissue remodeling (Gong et al. 2022; Millesi et al. 2023). In this study, The elevated FN1 expression in rat femoral bone scaffold at week 8 indicates active ECM organization, cell adhesion, and migration. The delayed peak of FN1 in nerve scaffold at week 12 suggests a slower yet focused regenerative trajectory. The chicken scaffold, despite its broad geometric surface, maintained moderate and relatively stable FN1 expression, implying a limited capacity for ECM stimulation.

Laminin facilitates cell migration, axonal growth, and synaptogenesis. In our study, the high LAMB2 expression in the rat femoral bone conduit reinforced its role in basal lamina reconstruction, cell migration, and axonal guidance. In contrast, the nerve scaffold displayed relatively low levels, possibly indicative of diminished lamina basal formation capacity. The chicken scaffold exhibited intermediate expression, potentially due to intrinsic ECM composition and physical scaffold characteristics.

Tenascin‐C is particularly important for axon pathfinding and is upregulated in regions of active neural repair (Ghayour et al., 2015. In this study, the peak in Tenascin‐C expression at week 1 in the chicken scaffold reflects an initial ECM response to neural injury, while the subsequent decline at week 4 across all groups marked the transition from Wallerian degeneration to the intermediate phase of regeneration). The second increase in Tenascin‐C expression at week 8, followed by the plateau from week 12 onward, corresponds to ECM stabilization and completion of reparative processes.

These molecular insights further support the regenerative potential of bone‐derived conduits.

As the second limitation of the current study, immunohistochemical quantification of inflammatory markers and cytokines or immune marker profiling that could have substantially improved the manuscript was not done in this study and can be suggested for future research.

Based on the findings of this study, acellular and demineralized bone conduits can:

1. Facilitate and accelerate reconnection between proximal and distal nerve stumps;
2. Support tissue‐level integration that may enable transmission of sensory and motor signals, though direct electrophysiological confirmation is needed; and
3. Enable neural signal conduction leading to functional recovery of the limb.

## 5 Conclusion

Considering the findings of the current study, bone‐derived conduits demonstrate strong potential as effective mediators in peripheral nerve repair. Further animal studies using alternative models are recommended. In subsequent phases, randomized clinical trials (RCTs) utilizing human CT‐derived conduits will be essential to evaluate their clinical efficacy.

## 6 Future Perspective

Future investigations should consider:

- long‐term assessments (> 6 months),
- integrated electrical or mechanical stimulation strategies,
- immunohistochemical profiling of vascularization and inflammation, and
- use of complex behavioral models, such as obstacle navigation.

## Author Contributions

**Zahra Sepehri**: investigation, writing – original draft, validation, visualization, formal analysis, data curation. **Nasser Mahdavi‐shahri**: conceptualization, writing – review and editing, validation, supervision. **Ali Moradi**: conceptualization, funding acquisition, supervision, resources, data curation, project administration, formal analysis, writing – review and editing, validation, visualization, methodology. **Morteza Behnam Rassouli**: conceptualization, writing – review and editing, supervision, project administration, resources, funding acquisition.

## Funding

This study was financially supported by Ferdowsi University of Mashhad (3/62617).

## Conflicts of Interest

The authors declare no conflicts of interest.

## Data Availability

Data are available upon request from the corresponding author.

## References

[brb371546-bib-0001] Abedin, E. , R. Lari , N. Mahdavi Shahri , and M. Fereidoni . 2018. “Development of a Demineralized and Decellularized Human Epiphyseal Bone Scaffold for Tissue Engineering: A Histological Study.” Tissue and Cell 55: 46–52. 30503059 10.1016/j.tice.2018.09.003

[brb371546-bib-0048] Ahmadi, F. , E. Hasanzadeh , A. Mellati , et al. 2025. “Multifunctional Electrospun PCL/CNT/EGCG Nerve Conduits With a Collagen Hydrogel for Enhanced Sciatic Nerve Regeneration.” Journal of Translational Medicine. 24, no. 1: 113. 41437258 10.1186/s12967-025-07561-5 PMC12837218

[brb371546-bib-0002] Anderson, J. M. , A. Rodriguez , and D. T. Chang . 2008. “Foreign Body Reaction to Biomaterials.” Seminars in Immunology 20, no. 2: 86–100. 18162407 10.1016/j.smim.2007.11.004 PMC2327202

[brb371546-bib-0003] Bain, J. R. , S. E. Mackinnon , and D. A. Hunter . 1989. “Functional Evaluation of Complete Sciatic, Peroneal, and Posterior Tibial Nerve Lesions in the Rat.” Plastic and Reconstructive Surgery 83, no. 1: 129–136. 2909054 10.1097/00006534-198901000-00024

[brb371546-bib-0004] Borschel, G. H. , K. F. Kia , W. M. Kuzon Jr , and R. G. Dennis . 2003. “Mechanical Properties of Acellular Peripheral Nerve.” Journal of Surgical Research 114, no. 2: 133–139. 14559438 10.1016/s0022-4804(03)00255-5

[brb371546-bib-0005] Carvalho, C. R. , W. Chang , J. Silva‐Correia , R. L. Reis , J. M. Oliveira , and J. Kohn . 2021. “Engineering Silk Fibroin‐Based Nerve Conduit With Neurotrophic Factors for Proximal Protection After Peripheral Nerve Injury.” Advanced Healthcare Materials 10, no. 2: e2000753. 33169544 10.1002/adhm.202000753

[brb371546-bib-0006] Dodla, M. C. , and R. V. Bellamkonda . 2006. “Anisotropic Scaffolds Facilitate Enhanced Neurite Extension in Vitro.” Journal of Biomedical Materials Research Part A 78A, no. 2: 213–221. 10.1002/jbm.a.30747 16892507

[brb371546-bib-0007] ElAbd, R. , A. Alabdulkarim , S. AlSabah , J. Hazan , B. Alhalabi , and S. Thibaudeau . 2022. “Role of Electrical Stimulation in Peripheral Nerve Regeneration: A Systematic Review.” Plastic and Reconstructive Surgery—Global Open 10, no. 3: e4115. 35317464 10.1097/GOX.0000000000004115 PMC8932473

[brb371546-bib-0008] Gao, Y. , Y. L. Wang , D. Kong , et al. 2015. “Nerve Autografts and Tissue‐Engineered Materials for the Repair of Peripheral Nerve Injuries: A 5‐Year Bibliometric Analysis.” Neural Regeneration Research 10, no. 6: 1003–1008. 26199621 10.4103/1673-5374.158369 PMC4498331

[brb371546-bib-0049] Ghayour, M. B. , A. Abdolmaleki , and M. Fereidoni . 2015. “Role of Extracellular Matrix in Peripheral Nerve Regeneration Process.” Razi Journal of Medical Sciences 135, no. 22: 75–88.

[brb371546-bib-0009] Gong, B. , X. Zhang , A. A. Zahrani , et al. 2022. “Neural Tissue Engineering: From Bioactive Scaffolds and in Situ Monitoring to Regeneration.” Exploration 2, no. 3: 20210035. 37323703 10.1002/EXP.20210035 PMC10190951

[brb371546-bib-0010] Gori, M. , G. Vadalà , S. M. Giannitelli , V. Denaro , and G. Di Pino . 2021. “Biomedical and Tissue Engineering Strategies to Control Foreign Body Reaction to Invasive Neural Electrodes.” Frontiers In Bioengineering and Biotechnology 9: 659033. 34113605 10.3389/fbioe.2021.659033 PMC8185207

[brb371546-bib-0011] Hargreaves, K. , R. Dubner , F. Brown , C. Flores , and J. Joris . 1988. “A New and Sensitive Method for Measuring Thermal Nociception in Cutaneous Hyperalgesia.” Pain 32, no. 1: 77–88. 3340425 10.1016/0304-3959(88)90026-7

[brb371546-bib-0012] Hatta, H. , K. Tsuneyama , K. Nomoto , et al. 2014. “A Simple and Rapid Decalcification Procedure of Skeletal Tissues for Pathology Using an Ultrasonic Cleaner With D‐Mannitol and Formic Acid.” Acta Histochemica 116, no. 5: 753–757. 24560938 10.1016/j.acthis.2014.01.006

[brb371546-bib-0050] Hu, Y.u , Y. Wu , Z. Gou , et al. 2016. “3D‐engineering of Cellularized Conduits for Peripheral Nerve Regeneration.” Scientific Reports. 6, no. 1: 32184. 27572698 10.1038/srep32184 PMC5004136

[brb371546-bib-0015] Jiang, T. , X.‐J. Ren , J.‐L. Tang , H. Yin , K.‐J. Wang , and C.‐L. Zhou . 2013. “Preparation and Characterization of Genipin‐Crosslinked Rat Acellular Spinal Cord Scaffolds.” Materials Science and Engineering: C 33, no. 6: 3514–3521. 23706241 10.1016/j.msec.2013.04.046

[brb371546-bib-0016] Jiang, X. , S. H. Lim , H. Q. Mao , and S. Y. Chew . 2010. “Current Applications and Future Perspectives of Artificial Nerve Conduits.” Experimental Neurology 223, no. 1: 86–101. 19769967 10.1016/j.expneurol.2009.09.009

[brb371546-bib-0017] Li, J. , Y. Liu , Y. Wang , and W. Xu . 2018. “Expression of Tenascin‐C in a Rat Vocal Fold Injury Model and Its Regulation of Fibroblasts.” The Laryngoscope 128, no. 9: E316–E322. 29572861 10.1002/lary.27164

[brb371546-bib-0018] Li, X. , X. Zhang , M. Hao , et al. 2022. “The Application of Collagen in the Repair of Peripheral Nerve Defect.” Frontiers in Bioengineering and Biotechnology 10: 973301. 36213073 10.3389/fbioe.2022.973301 PMC9542778

[brb371546-bib-0019] Lopes, B. , P. Sousa , R. Alvites , et al. 2022. “Peripheral Nerve Injury Treatments and Advances: One Health Perspective.” International Journal of Molecular Sciences 23, no. 2: 918. 35055104 10.3390/ijms23020918 PMC8779751

[brb371546-bib-0020] Lupon, E. , A. Acun , A. R. Andrews , et al. 2025. “A New Method for Preparation of Decellularized Human Scaffolds for Facial Reconstruction.” Current Issues in Molecular Biology 47, no. 4: 275. 40699674 10.3390/cimb47040275 PMC12026321

[brb371546-bib-0021] Mackinnon, S. E 1989. “Surgical Management of the Peripheral Nerve Gap.” Clinics in Plastic Surgery 16, no. 3: 587–603. 2673638

[brb371546-bib-0051] Mao, X. , T. Li , J. Cheng , et al. 2023. “Nerve ECM and PLA‐PCL Based Electrospun Bilayer Nerve Conduit for Nerve Regeneration.” Frontiers in Bioengineering and Biotechnology. 11: 2023. 10.3389/fbioe.2023.1103435 PMC10017983 36937756

[brb371546-bib-0022] Millesi, F. , S. Mero , L. Semmler , et al. 2023. “A Fibrous Nature of Hydrogels Promotes Directed Migration of Schwann Cells.” Plastic and Reconstructive Surgery Global Open 11: 3.

[brb371546-bib-0023] Montgomery, A. , J. Westphal , A. E. Bryan , and G. M. Harris . 2025. “Dynamically Changing Extracellular Matrix Stiffness Drives Schwann Cell Phenotype.” Matrix Biology Plus 25: 100167. 39868413 10.1016/j.mbplus.2024.100167 PMC11754676

[brb371546-bib-0024] Moore, A. M. , M. Macewan , K. B. Santosa , et al. 2011. “Acellular Nerve Allografts in Peripheral Nerve Regeneration: a Comparative Study.” Muscle & Nerve 44, no. 2: 221–234. 21660979 10.1002/mus.22033 PMC3136642

[brb371546-bib-0025] Mosmann, T. 1983. “Rapid Colorimetric Assay for Cellular Growth and Survival: Application to Proliferation and Cytotoxicity Assays.” Journal of Immunological Methods 65, no. 1–2: 55–63. 6606682 10.1016/0022-1759(83)90303-4

[brb371546-bib-0052] Nazeri, N. , M. A. Derakhshan , K. Mansoori , and H. Ghanbari . 2022. “Improvement of Sciatic Nerve Regeneration by Multichannel Nanofibrous Membrane‐embedded Electro‐conductive Conduits Functionalized With Laminin.” Journal of Materials Science: Materials in Medicine. 33, no. 6: 50. 35639181 10.1007/s10856-022-06669-0 PMC9156509

[brb371546-bib-0026] Petit, E. , V. Bavykina , M. Thibault , et al. 2024. “Assessing Tissue Mechanical Properties: Development of a Custom‐Made Tensile Device and Application on Rodents Sciatic Nerves.” Journal of the Mechanical Behavior of Biomedical Materials 159: 106709. 39216337 10.1016/j.jmbbm.2024.106709

[brb371546-bib-0029] Rahimi‐Movaghar, V. , A. Yazdi , and S. Saadat . 1970. “Saturated Picric Acid Prevents Autophagia.” Acta Medica Iranica 46, no. 4: 28–36.

[brb371546-bib-0030] Rbia, N. , L. F. Bulstra , P. F. Friedrich , A. T. Bishop , T. H. J. Nijhuis , and A. Y. Shin . 2020. “Gene Expression and Growth Factor Analysis in Early Nerve Regeneration Following Segmental Nerve Defect Reconstruction With a Mesenchymal Stromal Cell‐Enhanced Decellularized Nerve Allograft.” Plastic and Reconstructive Surgery—Global Open 8, no. 1: e2579. 32095395 10.1097/GOX.0000000000002579 PMC7015582

[brb371546-bib-0031] Sarker, M. , S. Naghieh , A. D. McInnes , D. J. Schreyer , and X. Chen . 2018. “Strategic Design and Fabrication of Nerve Guidance Conduits for Peripheral Nerve Regeneration.” Biotechnology Journal 13, no. 7: 1700635. 10.1002/biot.201700635 29396994

[brb371546-bib-0032] Shi, S. , X. Ou , and D. Cheng . 2023. “How Advancing Is Peripheral Nerve Regeneration Using Nanofiber Scaffolds? A Comprehensive Review of the Literature.” International Journal of Nanomedicine 18: 6763–6779. 38026517 10.2147/IJN.S436871 PMC10657550

[brb371546-bib-0033] Singh, V. K. , A. Haq , M. Tiwari , and A. K. Saxena . 2022. “Approach to Management of Nerve Gaps in Peripheral Nerve Injuries.” Injury 53, no. 4: 1308–1318. 35105440 10.1016/j.injury.2022.01.031

[brb371546-bib-0034] Summa Di, V. , P. J. Kingham , W. Raffoul , M. Wiberg , and G. Terenghi . 2011. “Adipose‐derived Stem Cells Enhance Peripheral Nerve Regeneration.” Journal of Plastic, Reconstructive & Aesthetic Surgery 64, no. 11: 1544–1551. 10.1016/j.bjps.2009.09.012 19828391

[brb371546-bib-0035] Syazwani, N. , A. Azhim , Y. Morimoto , K. S. Furukawa , and T. Ushida . 2015. “Decellularization of Aorta Tissue Using Sonication Treatment as Potential Scaffold for Vascular Tissue Engineering.” Journal of Medical and Biological Engineering 35, no. 2: 258–269.

[brb371546-bib-0036] Taifebagerlu, J. , and R. Mohammadi . 2015. “Effect of Local Administration of Brain Derived Neurotrophic Factor With Silicone Conduit on Peripheral Nerve Regeneration: A Rat Sciatic Nerve Transection Model.” Iranian Journal of Veterinary Surgery 10, no. 1: 43–52.

[brb371546-bib-0037] van Tonder, A. , A. M. Joubert , and A. D. Cromarty . 2015. “Limitations of the 3‐(4,5‐Dimethylthiazol‐2‐yl)‐2,5‐Diphenyl‐2H‐Tetrazolium Bromide (MTT) Assay When Compared to Three Commonly Used Cell Enumeration Assays.” BMC Research Notes 8, no. 1: 47. 25884200 10.1186/s13104-015-1000-8 PMC4349615

[brb371546-bib-0038] Wang, J.‐Y. , Y. Yuan , S.‐Y. Zhang , et al. 2024. “Remodeling of the Intra‐Conduit Inflammatory Microenvironment to Improve Peripheral Nerve Regeneration With a Neuromechanical Matching Protein‐Based Conduit.” Advanced Science 11, no. 17: 2302988. 38430538 10.1002/advs.202302988 PMC11077661

[brb371546-bib-0042] Yan, Y. , R. Yao , J. Zhao , et al. 2022. “Implantable Nerve Guidance Conduits: Material Combinations, Multi‐functional Strategies and Advanced Engineering Innovations.” Bioactive Materials 11: 57–76. 34938913 10.1016/j.bioactmat.2021.09.030 PMC8665266

[brb371546-bib-0053] Yu, W. , W. Zhao , C. Zhu , et al. 2011. “Sciatic Nerve Regeneration in Rats by a Promising Electrospun Collagen/Poly(ε‐caprolactone) Nerve Conduit With Tailored Degradation Rate.” BMC Neuroscience. 12, no. 1: 68. 21756368 10.1186/1471-2202-12-68 PMC3148572

[brb371546-bib-0043] Yun, C. , W. Li , Y. Qiao , et al. 2024. “Collagen Protein‐Chitosan Nerve Conduits With Neuroepithelial Stem Cells Promote Peripheral Nerve Regeneration.” Scientific Reports 14, no. 1: 20748. 39237597 10.1038/s41598-024-71435-x PMC11377726

[brb371546-bib-0044] Zarrintaj, P. , E. Zangene , S. Manouchehri , et al. 2020. “Conductive Biomaterials as Nerve Conduits: Recent Advances and Future Challenges.” Applied Materials Today 20: 100784.

[brb371546-bib-0045] Zhang, H. , J. Guo , Y. Wang , L. Shang , R. Chai , and Y. Zhao . 2022. “Natural Polymer‐Derived Bioscaffolds for Peripheral Nerve Regeneration.” Advanced Functional Materials 32, no. 41: 2203829.

[brb371546-bib-0046] Zhang, X. , T. Qi , Y. Sun , X. Cheng , P. Yang , and X. Dai . 2023. “Chitosan/hBMSC‐ECM Biomimetic Nerve Grafts Containing Orienting Microchannels for Peripheral Nerve Regeneration.” Biomaterials Advances 155: 213668. 39492002 10.1016/j.bioadv.2023.213668

[brb371546-bib-0047] Zhang, Z. , and M. Ma . 2024. “Strategies to Enhance the Ability of Nerve Guidance Conduits to Promote Directional Nerve Growth.” Biomedical Engineering Online 23, no. 1: 40. 38582838 10.1186/s12938-024-01233-z PMC10998375

[brb371546-bib-0054] Zheng, C. , Z. Yang , S. Chen , et al. 2021. “Nanofibrous Nerve Guidance Conduits Decorated With Decellularized Matrix Hydrogel Facilitate Peripheral Nerve Injury Repair.” Theranostics. 11, no. 6: 2917–2931. 33456580 10.7150/thno.50825 PMC7806490

